# Helicase Subunit Cdc45 Targets the Checkpoint Kinase Rad53 to Both Replication Initiation and Elongation Complexes after Fork Stalling

**DOI:** 10.1016/j.molcel.2018.11.025

**Published:** 2019-02-07

**Authors:** Geylani Can, Anastasia Christine Kauerhof, Dominik Macak, Philip Zegerman

**Affiliations:** 1 Wellcome Trust/Cancer Research UK Gurdon Institute and Department of Biochemistry, The Henry Wellcome Building of Cancer and Developmental Biology, University of Cambridge, Cambridge CB2 1QN, UK

**Keywords:** DNA replication, checkpoint, Rad53, DNA damage, replisome, Cdc45, budding yeast

## Abstract

Across eukaryotes, disruption of DNA replication causes an S phase checkpoint response, which regulates multiple processes, including inhibition of replication initiation and fork stabilization. How these events are coordinated remains poorly understood. Here, we show that the replicative helicase component Cdc45 targets the checkpoint kinase Rad53 to distinct replication complexes in the budding yeast *Saccharomyces cerevisiae*. Rad53 binds to forkhead-associated (FHA) interaction motifs in an unstructured loop region of Cdc45, which is phosphorylated by Rad53 itself, and this interaction is necessary for the inhibition of origin firing through Sld3. Cdc45 also recruits Rad53 to stalled replication forks, which we demonstrate is important for the response to replication stress. Finally, we show that a Cdc45 mutation found in patients with Meier-Gorlin syndrome disrupts the functional interaction with Rad53 in yeast. Together, we present a single mechanism by which a checkpoint kinase targets replication initiation and elongation complexes, which may be relevant to human disease.

## Highlights

• Cdc45 targets Rad53 to inhibit replication initiation through Sld3
• Rad53 binds to the replisome in part through Cdc45
• A Meier-Gorlin mutation in Cdc45 prevents interaction with Rad53

## Graphical Abstract

**Figure:**
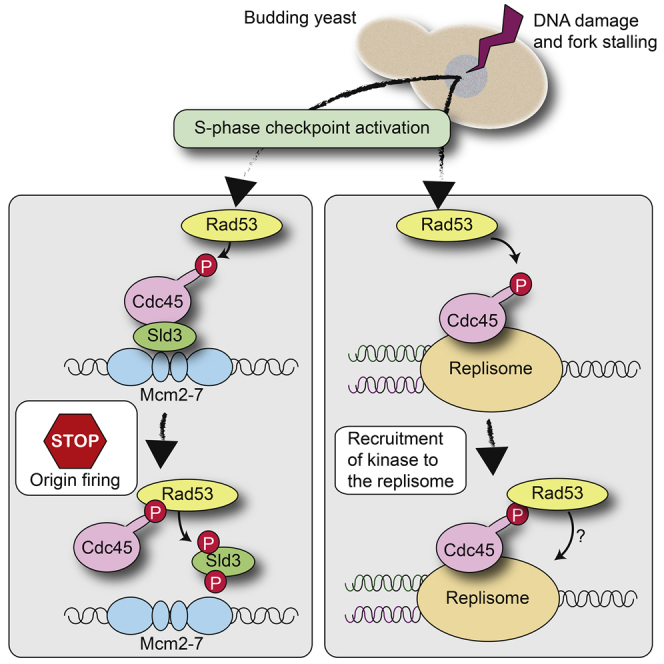

## Teaser

Checkpoint kinases are critical for multiple responses to DNA damage. Can et al. show that the Rad53 kinase is targeted to regulate both replication initiation and stalled replisomes through an interaction with the helicase subunit Cdc45. This provides a single mechanism that targets this kinase to regulate distinct checkpoint events.

## Introduction

Eukaryotic DNA replication is tightly regulated to ensure that the genome is replicated in its entirety in every cell cycle. The first step in replication involves the formation of the pre-replicative complex (pre-RC) at origins in G1 phase, a process called “licensing” (Remus and Diffley, 2009). The licensing reaction results in the loading of inactive double hexamers of the Mcm2–7 helicase on double-stranded DNA. Subsequently, replication initiation at licensed origins can only occur in S phase due to the activation of the S-phase cyclin-dependent kinase (S-CDK) and Dbf4-dependent (DDK) kinases (Labib, 2010).

DDK directly phosphorylates the inactive Mcm2–7 double hexamers, resulting in structural alterations (Sheu and Stillman, 2010) and the generation of a binding site for the initiation factors Sld3 and Sld7 (Deegan et al., 2016). Sld3 binding to Mcm2–7 facilitates the recruitment of Cdc45, which is a critical component of the active form of the replicative helicase, called the CMG (Cdc45-Mcm-GINS) complex (Bell and Labib, 2016). Cyclin-dependent kinase (CDK), on the other hand, phosphorylates Sld3 and an additional initiation factor, Sld2, which via phospho-interactions with Dpb11 results in the recruitment of GINS and the leading-strand polymerase (Pol ε) to origins (Tanaka and Araki, 2013). Together, DDK and CDK are required both for the ordered assembly of the active CMG complex and for the recruitment of additional proteins to form the multi-subunit replication machinery, called the replisome (Tanaka and Araki, 2013).

DNA lesions or low levels of deoxynucleotide triphosphates (dNTPs) cause stalling of the replisome during DNA synthesis, leading to the exposure of single-stranded DNA at the replication fork and activation of the checkpoint kinases ATR and Mec1 (in humans and budding yeast, respectively; Saldivar et al., 2017). In conjunction with a mediator protein (Claspin/Mrc1) that binds to the replisome (Errico and Costanzo, 2012), activation of ATR and Mec1 leads to the activation of the effector kinase Chk1 in humans or Rad53 in yeast (Giannattasio and Branzei, 2017). This response to fork stalling is called the S phase, intra-S phase, or DNA replication checkpoint (Pardo et al., 2017).

The S phase checkpoint results in a range of responses, including the upregulation of dNTPs and the coordination of DNA repair (Giannattasio and Branzei, 2017, Labib and De Piccoli, 2011, Saldivar et al., 2017). In addition, this checkpoint also directly regulates DNA replication itself. First of all, checkpoint activation in S phase results in the inhibition of further origin firing (Zegerman and Diffley, 2009). In budding yeast, Rad53 phosphorylates and inhibits two replication initiation factors, Dbf4 and Sld3 (Lopez-Mosqueda et al., 2010, Zegerman and Diffley, 2010). Although it is not clear how Rad53 inhibits Dbf4, phosphorylation of Sld3 by Rad53 inhibits its interactions with Mcm2–7, Dpb11, and Cdc45 (Deegan et al., 2016, Lopez-Mosqueda et al., 2010, Zegerman and Diffley, 2010).

In addition to regulating origin firing, the checkpoint response enables forks to resume replication after stalling in a process called fork stabilization (Giannattasio and Branzei, 2017, Labib and De Piccoli, 2011, Saldivar et al., 2017). In cells that lack checkpoint activity, replication forks cannot continue DNA synthesis after stalling; DNA unwinding and DNA synthesis become uncoupled (Gan et al., 2017), and the fork is said to have collapsed (Giannattasio and Branzei, 2017, Saldivar et al., 2017), although the replisome itself remains largely intact under these conditions (De Piccoli et al., 2012, Dungrawala et al., 2015). How the active checkpoint kinases specifically target replication complexes after DNA damage and fork stalling remains poorly understood.

Here, we demonstrate a mechanism that recruits the active checkpoint kinase Rad53 to replication complexes in budding yeast. We show that the replication initiation and elongation factor Cdc45 targets Rad53 to Sld3 to inhibit origin firing, and when incorporated into the replisome as part of the CMG complex, it also recruits Rad53 to stabilize stalled forks. This study provides a single mechanism that coordinates the checkpoint regulation of both replication initiation and fork stalling after replication stress *in vivo*.

## Results

### Cdc45 Mediates Rad53-Dependent Sld3 Phosphorylation *In Vivo*

**Figure 1:**
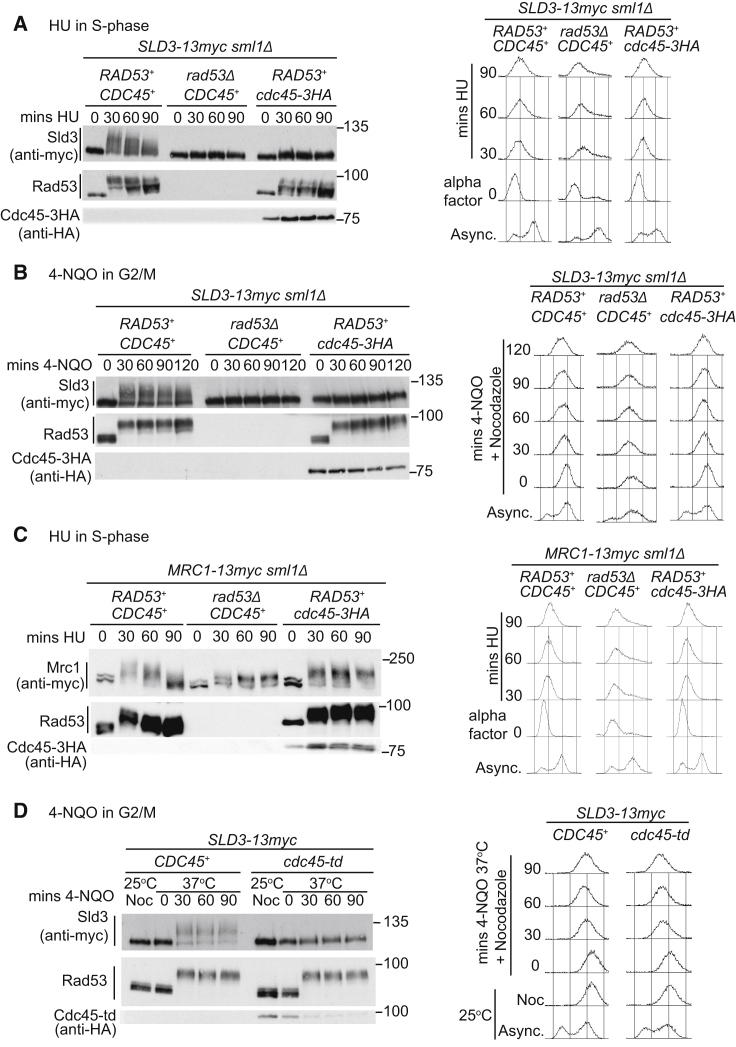
Cdc45 Is Required for Rad53-Dependent Phosphorylation of Sld3 *In Vivo* (A) Western blots (left) and flow cytometry (right) of the indicated yeast strains released from G1 arrest in alpha factor (time 0) into 200 mM HU. *sml1Δ* is required for the viability of *rad53Δ* strains. (B) As in (A), except strains were arrested in nocodazole and treated with 10 μg/mL 4-NQO. (C) As in (A), except that Mrc1–13myc was resolved on a PhosTag gel. (D) As in (B), except strains were first arrested at 25°C and then shifted to 37°C before addition of 4-NQO.

We have previously shown that activation of Rad53 in budding yeast (e.g., after treatment with hydroxyurea [HU], which causes global fork stalling) results in phosphorylation of the replication initiation factor Sld3 (Zegerman and Diffley, 2010). We observed, however, that the Rad53-dependent phosphorylation of Sld3 was largely abrogated in strains containing a tagged allele of the helicase co-factor *CDC45* (*cdc45-3HA*; Figure 1A). This allele of *CDC45* is not fully functional (a hypomorph), resulting in reduced origin firing, a slower S phase, and synthetic lethality with checkpoint mutants (Figures S1A–S1C). The *cdc45-3HA* allele also showed reduced Rad53 activation during S phase, as detected by the abundance of the phosphorylated forms of Rad53 (Figure 1A), which is likely because defects in replication initiation have a knock-on effect on the number of stalled forks (Tercero et al., 2003). Loss of Sld3 phosphorylation in the *cdc45-3HA* strain was not simply a consequence of reduced Rad53 activation, however, as we observed the same effect after DNA damage in G2/M-arrested cells, where Rad53 activation is independent of DNA replication and unaffected by the *cdc45-3HA* allele (Figure 1B). To further show that *cdc45-3HA* has a specific defect in Rad53-dependent Sld3 phosphorylation, we also analyzed other Rad53 targets. The Rad53-dependent phosphorylation of both Dbf4 and the checkpoint mediator protein Mrc1 was very similar to wild-type in the presence of Cdc45-3HA (Figures 1C and S1E), unlike the situation for Sld3. Therefore, we conclude that Cdc45-3HA has a specific defect in Rad53-dependent phosphorylation of Sld3.

Since *cdc45-3HA* is a hypomorph, we wondered whether the abrogation of Sld3 phosphorylation was due to a loss of function of Cdc45. If this were the case, then a null allele of *CDC45* should phenocopy the *cdc45-3HA* mutant. As *CDC45* is an essential gene, we used a temperature-sensitive *cdc45* degron allele (*cdc45-td*) to test this hypothesis. As with *cdc45-3HA*, *cdc45-td* resulted in a dramatic reduction in DNA-damage-dependent Sld3 phosphorylation (Figure 1D). This effect was specific to loss of Cdc45, as loss of function of Dpb11, another Sld3-interacting protein, did not alter the phosphorylation of Sld3 (Figure S2A). Together, these data show that Cdc45 is required for Rad53-dependent phosphorylation of Sld3 *in vivo*.

### A Rad53 Interaction Mutant of Cdc45 Prevents Sld3 Phosphorylation *In Vivo*

**Figure 2:**
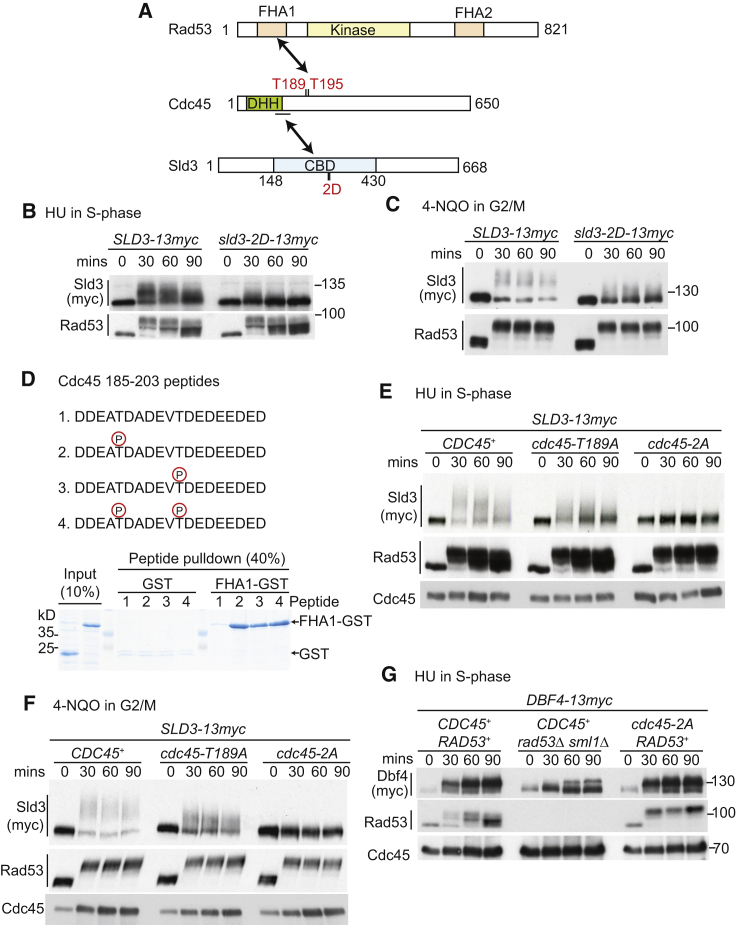
Interactions between Sld3, Cdc45, and Rad53 Are Required for Rad53-Dependent Phosphorylation of Sld3 (A) Scale diagram of budding yeast Rad53, Cdc45, and Sld3. The Cdc45 DHH phosphoesterase homology domain is in green. The Cdc45-binding domain (CBD) of Sld3 is in blue. The 2D mutant refers to Sld3 S306D and T310D. (B, E, and G) Western blots of the indicated strains, released from G1 arrest in alpha factor (time 0) into 200mM HU. (C and F) Western blots of the indicated strains arrested in nocodazole (0) and treated with 10 μg/ml 4-NQO for the indicated times. (D) The indicated Cdc45 peptides were immobilized on beads and used in pull-down assays with glutathione S-transferase (GST) or Rad53-FHA1-GST. A Coomassie stained gel of the indicated percentage of input and pull-down is shown.

As Cdc45 is known to bind to Sld3, we wondered whether the phenotypes of *cdc45-3HA* might be due to reduced interaction of this tagged protein with Sld3. Although Cdc45-3HA and wild-type protein were expressed at similar levels (Figure S2B), we observed by yeast-two-hybrid analysis that Cdc45-3HA interacted less well with Sld3 (Figure S2C), which might explain the replication defects associated with this allele (Figures S1A–S1C). To further explore whether a Cdc45-Sld3 interaction is required for Rad53-dependent phosphorylation of Sld3, we analyzed a mutant of Sld3 (*sld3-2D*; Figure 2A) that has reduced binding to Cdc45 (Zegerman and Diffley, 2010). Significantly, as with the *cdc45-3HA* allele, *sld3-2D* exhibited a dramatic reduction in Sld3 phosphorylation (Figure 2B) that was not due to defects in Rad53 activation since this effect was also observed after DNA damage in G2/M arrested cells (Figure 2C). The reduction in Sld3 phosphorylation in both the *cdc45-3HA* and *sld3-2D* mutants suggests that the Cdc45-Sld3 interaction is important for Rad53 targeting of Sld3.

A previous study, screening for interacting partners of the FHA1 domain of Rad53, identified a specific interaction between Rad53 and Cdc45 (Aucher et al., 2010). This suggested that Cdc45 might facilitate Sld3 phosphorylation by bridging Rad53 and Sld3 (Figure 2A). The interaction site between Cdc45 and the FHA1 domain of Rad53 was shown to be within a poorly conserved acidic loop region of Cdc45 (Aucher et al., 2010), which contains two canonical forkhead-associated (FHA) interaction motifs, pTxxD (where pT is phospho-threonine) starting at codons 189 and 195 (Figures 2A and S2D). To confirm that these sites directly interact with FHA1 of Rad53, we performed peptide pull-down experiments using purified proteins. Phosphorylation of T189 or T195 or both was sufficient to bind to the Rad53 FHA1 domain (Figure 2D). In addition, mutation of T189 and T195 to alanine (hereafter called *cdc45-2A*) prevented the interaction with Rad53 (Figure S2E). Therefore, in line with a previous study (Aucher et al., 2010), phosphorylation of Cdc45 T189 and T195 generates binding sites for the FHA1 domain of Rad53.

To test whether these Cdc45-Rad53 interaction sites are required for Sld3 phosphorylation *in vivo*, we replaced the wild-type copy of *CDC45* with *cdc45-2A*. While the T189A mutation alone caused a reduction in Sld3 phosphorylation, mutation of both T189 and T195 (*cdc45-2A*) resulted in a dramatic loss of Sld3 phosphorylation *in vivo* both in S phase in HU (Figure 2E) and in G2/M phase in 4-NQO (Figure 2F). This effect was specific for Sld3, as *cdc45-2A* did not impact the Rad53-dependent phosphorylation of Dbf4 or Mrc1 (Figures 2G and S2F). Unlike *cdc45-3HA*, the *cdc45-2A* allele did not affect S phase dynamics (Figure S1A), was not synthetic lethal with checkpoint mutants (Figure S1D), and did not result in reduced Rad53 activation in S phase (Figures 2E and 2G). Therefore, while the *cdc45-2A* allele showed reduced interaction with Rad53 (Figure S2E) and largely abrogated Rad53-dependent Sld3 phosphorylation (Figures 2E and 2F), we did not detect any defects in the essential functions of Cdc45 associated with the *2A* allele. Together, these genetic analyses show that mutants that interfere with the Cdc45-Sld3 interaction or the Cdc45-Rad53 interaction (Figure 2A) are sufficient to abrogate Rad53 phosphorylation of Sld3 *in vivo*.

### The Cdc45-Rad53 Interaction Is Required for Inhibition of Sld3

**Figure 3:**
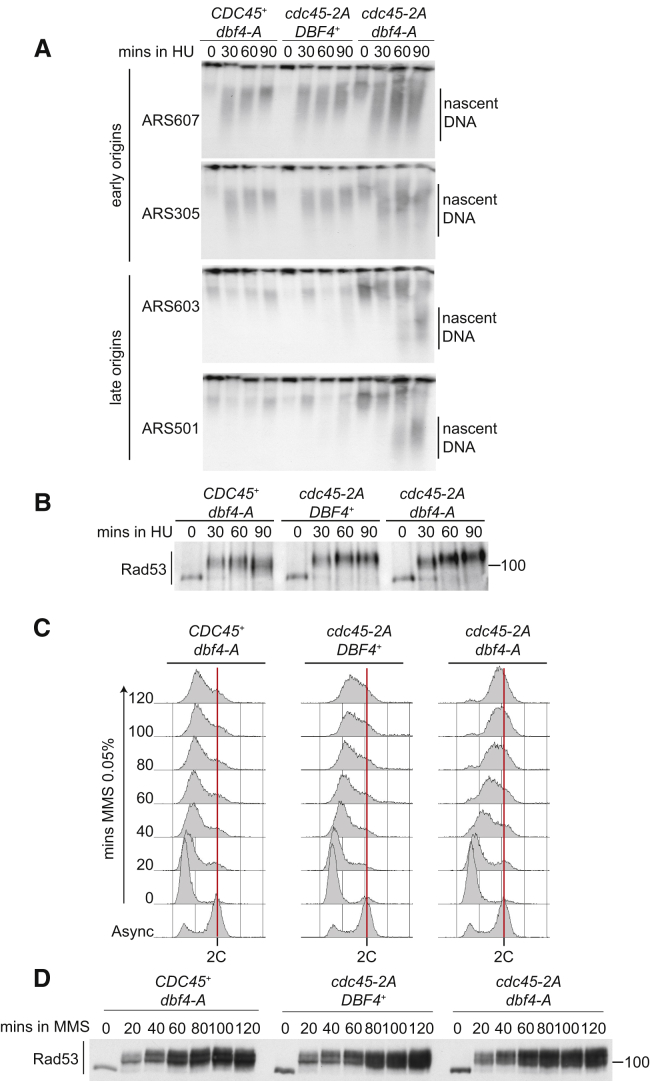
The Cdc45-Rad53 Interaction Is Required for Inhibition of Sld3 Function (A) Autoradiograms of Southern blots of alkaline gels showing replication intermediates from the indicated strains released from alpha factor (time 0) into 200 mM HU at 25°C. (B) Anti-Rad53 western blot of the experiment in (A). (C) Flow cytometry of the indicated strains arrested in G1 with alpha factor and released into 0.05% MMS. (D) Anti-Rad53 western blot of the experiment in (C).

In budding yeast, Rad53 blocks replication initiation by inhibiting both Sld3 and Dbf4 (Lopez-Mosqueda et al., 2010, Zegerman and Diffley, 2010). Mutating the Rad53 phosphorylation sites in Sld3 and Dbf4 to alanine (hereafter called the *sld3-A* and *dbf4-A* alleles) is sufficient to alleviate the block to origin firing in HU and allows fast progression through S phase in the presence of the DNA alkylating agent methyl methanesulfonate (MMS) (Zegerman and Diffley, 2010). We therefore reasoned that if the Cdc45-Rad53 interaction mutant (*cdc45-2A*) prevents the inhibitory phosphorylation of Sld3, then *cdc45-2A* together with *dbf4-A* should also be sufficient to derepress origin firing after checkpoint activation. To test this, we arrested yeast cells in G1 phase and released them into S phase in the presence of HU or MMS. The *dbf4-A* mutant alone and the *cdc45-2A* mutant alone did not result in the appearance of nascent DNA at late-firing origins in HU (Figure 3A), nor did they significantly accelerate S phase progression in MMS (Figure 3C). Importantly, however, when *dbf4-A* was combined with *cdc45-2A*, nascent DNA was observed at late-firing origins (Figure 3A), and the cells rapidly progressed through S phase (Figure 3C), similar to a *RAD53* null strain (Zegerman and Diffley, 2010). This effect of *cdc45-2A/dbf4-A* on origin firing and S phase progression was not due to defects in Rad53 activation (Figures 3B and 3D). From this experiment, we conclude that the Rad53-Cdc45 interaction is required for the phosphorylation and inhibition of Sld3 in the presence of DNA damage.

### Cdc45 Enhances Rad53-Dependent Sld3 Phosphorylation *In Vitro* and Is Phosphorylated by Rad53 *In Vivo*

**Figure 4:**
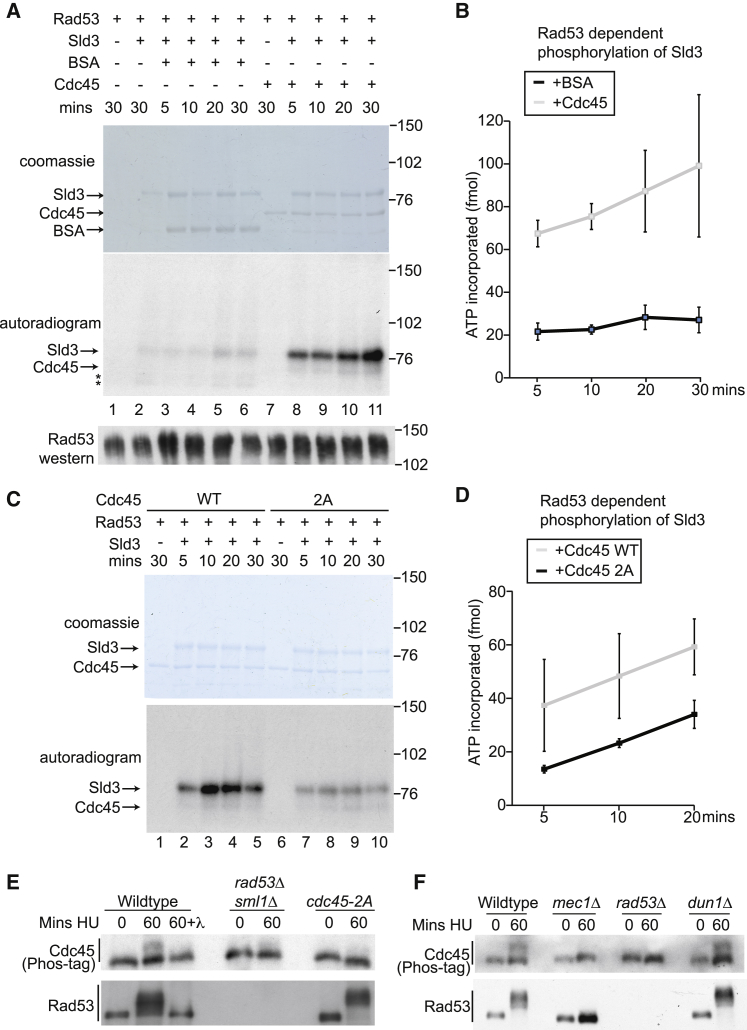
Cdc45 Directly Enhances Rad53-Dependent Sld3 Phosphorylation *In Vitro* and Is Phosphorylated by Rad53 *In Vivo* (A) Kinase assay using Rad53, Sld3, and either BSA or Cdc45. Sld3, Cdc45, and BSA were in 25-fold excess over Rad53. Top: Coomassie-stained gel. Middle: autoradiogram. Bottom: Rad53 western blot. Asterisks mark degradation products of Sld3. (B) Quantitation of the kinase assay in (A). Error bars represent SD; n = 3. (C) As in (A), except using either wild-type Cdc45 or the Cdc45-2A mutant. (D) As in (B). (E) Western blot of the indicated strains released from alpha factor (time 0) into 200 mM HU. The Cdc45 western blot was performed using PhosTag PAGE. 60+λ is the addition of λ phosphatase to the 60-min HU time point. (F) As in (E).

To demonstrate a direct role for Cdc45 in the Rad53-dependent phosphorylation of Sld3, we set out to recapitulate this pathway *in vitro* using only bacterially purified proteins. We have previously shown that Sld3 is a target of Rad53 *in vitro* (Zegerman and Diffley, 2010), but significantly, when we added Cdc45 to this reaction in an equimolar ratio to Sld3, we observed considerable enhancement of Rad53-dependent Sld3 phosphorylation (Figures 4A and 4B), consistent with our results *in vivo*. To exclude the possibility that Cdc45 protein stimulates Rad53 activity nonspecifically, we performed the same reaction except using a C-terminal fragment of Sld3 (530–668). This fragment of Sld3 contains many Rad53 sites, which are phosphorylated *in vitro* and *in vivo* (Zegerman and Diffley, 2010), but does not contain the Cdc45 interaction domain (Figure 2A). Importantly, we observed no stimulation of Rad53 phosphorylation of Sld3 530–668 in the presence of Cdc45 (Figure S3A), demonstrating that Cdc45 is not simply enhancing Rad53 activity nonspecifically.

Since the Cdc45-2A mutant showed reduced interaction with Rad53 (Figure S2E) and reduced phosphorylation of Sld3 *in vivo* (Figures 2E and 2F), we wondered whether this mutant would affect Sld3 phosphorylation by Rad53 *in vitro*. Indeed, compared to Cdc45 wild-type protein, the Cdc45-2A mutant showed reduced stimulation of Sld3 phosphorylation *in vitro* (Figures 4C and 4D), consistent with the *in vivo* results.

As phosphorylation of Cdc45 is required for the Rad53 interaction (Figure 2D) yet Cdc45 purified from bacteria is sufficient to stimulate Sld3 phosphorylation (Figure 4A), we wondered whether the T189 and T195 residues in Cdc45 might become phosphorylated in this assay. While Sld3 is a very efficient substrate of Rad53, as it contains at least 38 Rad53 phosphorylation sites (Zegerman and Diffley, 2010), we did also observe Rad53-dependent Cdc45 phosphorylation *in vitro* (Figures 4A, 4C, S3A, and S3B). Interestingly, the Cdc45-2A mutant was less phosphorylated than the wild-type Cdc45 protein *in vitro* (Figure S3C), suggesting that these sites can be directly phosphorylated by Rad53.

Since Rad53 can phosphorylate Cdc45 *in vitro*, we wondered whether this might be the case *in vivo*. To test the physiological phospho-status of Cdc45, we analyzed yeast extracts on phos-tag gels. Cdc45 exhibited a lower-mobility form in S phase cells treated with HU, which was sensitive to phosphatase treatment (Figure 4E). This Cdc45 phosphorylation was dependent on Rad53 and the upstream checkpoint kinase Mec1, but not the downstream kinase Dun1 (Figures 4E and 4F). Importantly, this phospho-shift of Cdc45 was also largely abrogated in the Cdc45-2A mutant (Figure 4E), suggesting that residues T189 and T195 are phosphorylated by Rad53 *in vivo*. From Figure 4, we conclude that Cdc45 is sufficient to directly stimulate Rad53-dependent phosphorylation of Sld3 and that Rad53 mediates the phosphorylation of Cdc45 *in vivo*.

### Cdc45 Targets Rad53 to Replication Forks

Cdc45 binds to Sld3 during replication initiation and is also incorporated into the replisome as a component of the CMG helicase (Bell and Labib, 2016). Structural analysis has shown that the flexible acidic loop of Cdc45, which binds to Rad53 in yeast (Figures 2D and S2E), protrudes from the CMG complex, away from the Mcm2–7 and GINS interfaces (Simon et al., 2016). Therefore, we wondered whether this flexible loop of Cdc45 might not only allow Rad53 to bind Sld3 to inhibit replication initiation but also mediate interactions between Rad53 and the replisome. To test this, we set out to analyze interactions between Rad53 and stalled replisomes by chromatin immunoprecipitation (ChIP).

Before comparing the Rad53 ChIP signal at forks between strains, we first addressed whether the *cdc45-2A* mutant affects the position of replication forks in HU. For this, we used ChIP of polymerase alpha subunit Pol1 as previously described (De Piccoli et al., 2012). By aligning the ChIP signal at all yeast origins according to their normal time of replication (t_rep_; Raghuraman et al., 2001), the position of Pol1 indicated that in the *cdc45-2A* strain replisomes formed at more late-firing origins and moved less far than in the *CDC45* wild-type strain (Figure S4A). The altered position of the replication machinery in the *cdc45-2A* strain was also confirmed by the detection of replicated DNA through copy-number analysis (Figure S4B). As the Cdc45-2A mutant prevents the inhibition of Sld3 by Rad53 in HU (Figure 2E), our interpretation of this Pol1 ChIP is that Cdc45-2A, by failing to inhibit Sld3, allows slightly more late-origin firing than in a wild-type strain in HU. This extra origin firing potentially results in a faster depletion of nucleotide pools, causing the replisomes that are formed at early origins to stall even closer to the origin, as previously observed (Zegerman and Diffley, 2010). Indeed, this small effect of Cdc45-2A alone on late-origin firing might also explain the observation that in MMS, this mutant has a slightly faster S phase than the wild-type strain (Figure 3C) and shows increased Rad53 activation in HU (e.g., Figures 2E and S2F). As the Cdc45-2A mutant affects the location of replisomes in HU, this presented a challenge for the direct comparison of the Rad53 ChIP signal between *cdc45-2A* and wild-type strains.

To circumvent differences in origin firing between strains, we utilized the *sld3-A* and *dbf4-A* alleles, which cannot be inhibited by Rad53 and therefore allow efficient origin firing genome-wide in HU (Zegerman and Diffley, 2010). Indeed, analysis of DNA copy number shows that the *sld3-A dbf4-A* mutants facilitated origin firing efficiently at both early and late origins, regardless of the presence of the *cdc45-2A* mutant (Figure S4C). Since the *sld3-A* and *dbf4-A* alleles ensure that origin firing and subsequently replisome position is the same between strains (Figure S4C), this allowed a direct comparison of fork proteins by ChIP.

To allow normalization between Rad53 ChIP sequencing samples, we utilized an allele of the centromeric histone Cse4 containing the same tag as Rad53 (hemagglutinin [HA]) to act as an internal standard (Figures S5A and S5B). Importantly, HA-Rad53 did not affect the enrichment of Cse4-HA ChIP at centromeres (Figure S5C), showing that it is an effective internal standard for Rad53 ChIP. As a result, we could directly compare between the datasets using the average ChIP signal within 1 kb of all centromeres to scale the sequencing reads between strains (Figure S5B).

**Figure 5:**
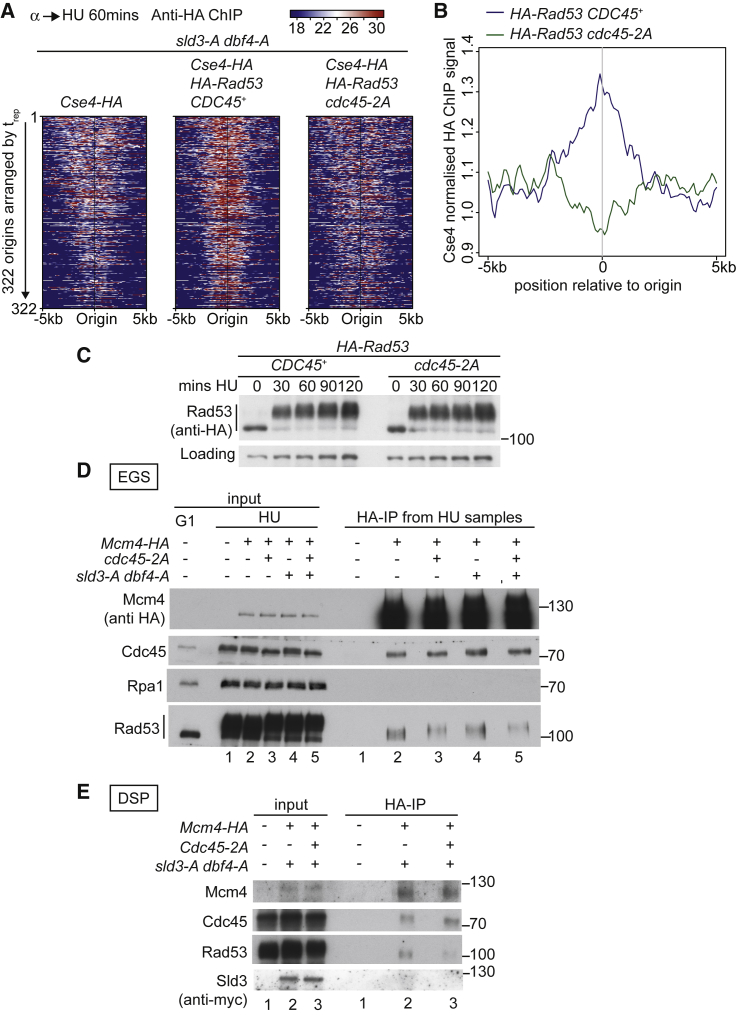
Cdc45 Targets Rad53 to Replication Forks (A) Anti-HA ChIP of the indicated strains released from alpha factor (G1) into 200 mM HU for 60 min. Data are presented as a heatmap of sequencing reads normalized to the reads from 500 bp on either side of all yeast centromeres. The map is centered on 322 of the yeast origins, arranged by increasing t_rep_. (B) Data from (A) were normalized to the reads from Cse4-HA-tagged strain alone. Graph represents an average signal from 322 origins, centered on the origin. (C) Rad53 western blot from indicated strains as in Figure 1A. (D) Immunoprecipitation of Mcm4-HA in the presence of the crosslinking agent EGS from the indicated strains released from alpha factor (G1) into 200 mM HU for 60 min. (E) As in (D), except performed in the presence of DSP, not EGS. The *sld3-A* allele is Myc-tagged.

Using *sld3-A dbf4-A* to ensure that all origins fire equally and Cse4-HA to allow normalization of our ChIP-sequencing reads, we performed ChIP of HA-Rad53 in Cdc45 wild-type and mutant strains. Previous attempts to ChIP Rad53 at forks resulted in only very weak signal (Katou et al., 2003), so we used both formaldehyde and the 16-Å ethylene glycol bis(succinimidyl succinate) (EGS) crosslinker to capture more interactions. Using this method, we observed a ChIP signal around all origins in the HA-Rad53-tagged strain with wild-type Cdc45, but not in the untagged Rad53 control strain (Figure 5A, left versus middle heatmap). Importantly, in the strain with the *cdc45-2A* mutation, we observed only a background level of ChIP signal (Figure 5A, right heatmap). To ensure that we only compared the ChIP signal from HA-Rad53 we normalized the ChIP data to the reads from the Cse4-HA strain alone (Figure 5A, left heatmap) and represented the data as an average signal at all origins (Figure 5B). It is clear from this analysis that the specific ChIP signal for Rad53 around origins was greatly reduced in the *cdc45-2A* mutant. Instead of normalizing to Cse4-HA, we also obtained a similar result when the data were normalized to the background reads at an unreplicated locus (Figure S5D), demonstrating that Figure 5B is not an artifact of Cse4 normalization.

Several lines of evidence indicate that this Rad53 ChIP signal, although centered on origins, is at stalled forks and not at unfired origins. First, almost all origins fire efficiently in the *sld3-A dbf4-A* strain (Figure S4C). Second, the Rad53 ChIP reaches up to 2 kb away from the origins, in line with the position of replicated DNA (e.g., Figure S4C). Third, in a longer time course in HU, the Rad53 ChIP signal moved with the fork position (Figure S5E). From these ChIP analyses, we conclude that Cdc45’s interaction with Rad53 is important to recruit Rad53 to stalled forks.

Although *cdc45-2A* had a dramatic effect on Rad53 recruitment to forks in this ChIP assay, we observed no change in Rad53 activation under these conditions (Figure 5C). This indicates that the Cdc45-dependent mechanism of recruitment of Rad53 to the replisome does not affect Rad53 activation (see Discussion).

We noticed that tagging Rad53 reduced the levels of Rad53 protein (Figures S6A and S6B), in line with previous studies (Conde et al., 2010). As the levels of Rad53 might affect the total ChIP signal, we decided to analyze the interaction of wild-type untagged Rad53 with replisomes. To capture transient Rad53 interactions with the replisome, we performed immunoprecipitation (IP) of Mcm4 in the presence of the crosslinking agents EGS or dithiobis(succinimidyl propionate) (DSP) (Figures 5D and 5E). As expected, IP of Mcm4-HA from HU-arrested yeast extracts resulted in coIP of Cdc45 (Figures 5D and 5E). The single-stranded DNA (ssDNA)-binding protein Rpa1, which is not in direct contact with the CMG complex, was not present in this IP, showing that the interactions are specific (Figure 5D). Importantly, Rad53 was precipitated specifically in the Mcm4-tagged strain, but not in an untagged control (Figures 5D and 5E, compare IP 1 and IP 2). This interaction was mediated at least partly by the flexible loop of Cdc45, as the Cdc45-2A mutant, while not affecting the interaction between Cdc45 and Mcm4, resulted in a 40%–50% reduction in the interaction with Rad53 (Figure 5D, compare IP 2 and IP 3).

To rule out that this IP was detecting initiation complexes at loaded Mcm2–7 double hexamers rather than the active CMG complex, we also conducted the experiment with strains containing the *sld3-A dbf4-A* alleles, which allow almost all origins to fire in HU (Figure S4C). Even when all origins fire, we still observed an interaction between Rad53 and Mcm4, which was reduced in the Cdc45-2A mutant (Figure 5D, compare IP 4 and IP 5, and Figure 5E, compare IP 2 and IP 3). We did not detect any Sld3 in this IP (Figure 5E), again confirming that these IPs are specific for the CMG complex not for unfired origins. The qualitative difference in the amount of residual Rad53 interacting with replisomes in the *cdc45-2A* strain as detected by ChIP versus coIP (compare Figure 5B with Figures 5D and 5E) may be a reflection of the reduced expression of HA-Rad53 in the ChIP strain or the limitation of ChIP for identifying interactions that are distal to the DNA. Despite this, Figure 5 shows that Rad53 interacts with the replisome and that this is at least partially dependent on its binding sites (T189 and T195) in Cdc45.

### Cdc45’s Interaction with Rad53 Is Required for Viability During Fork Stalling

A critical function of Rad53 is to stabilize replication forks after stalling (Tercero et al., 2003). Despite this, the *cdc45-2A* mutant that has defects in the recruitment of Rad53 to the replisome (Figure 5) showed very little loss of viability in a range of DNA-damaging agents (Figure S6C). We therefore wondered whether additional pathways that can also recruit Rad53 to the replisome might be redundant with the Cdc45-dependent recruitment mechanism.

**Figure 6:**
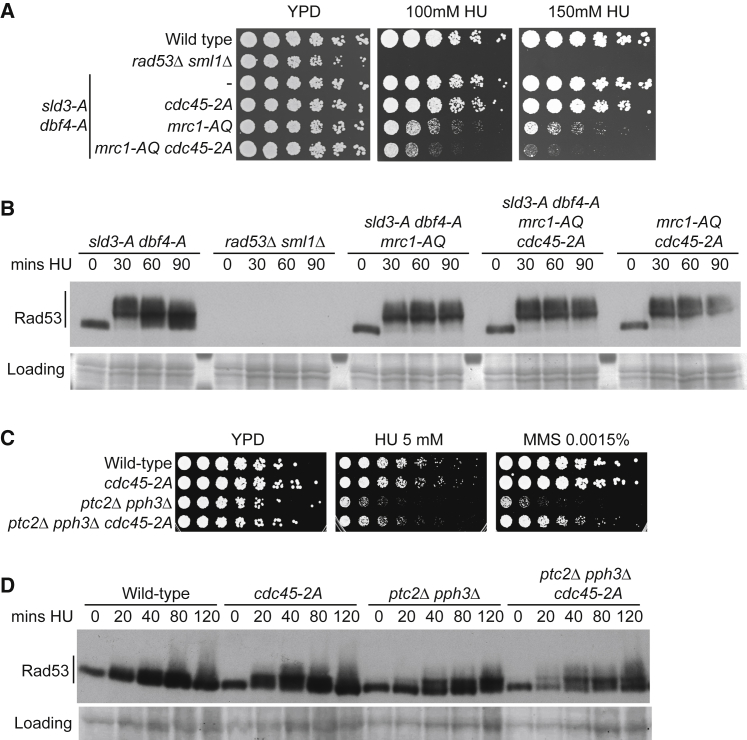
Cdc45’s Interaction with Rad53 Is Required for Viability during Fork Stalling (A) Growth assay of the indicated strains. The lower 4 strains all contain the *sld3-A* and *dbf4-A* alleles. (B) Anti-Rad53 western blot of the indicated strains released from alpha factor (time 0) into 200 mM HU. Loading control is a section of the Ponceau-stained nitrocellulose membrane. (C) As in (A). (D) As in (B).

One replisome protein that is known to interact with Rad53 is the checkpoint mediator protein Mrc1. Phosphorylation of Mrc1 by Mec1 generates binding sites for the N-terminal FHA domain of Rad53 (Chen and Zhou, 2009, Tanaka and Russell, 2004). To test a role for both Mrc1 and Cdc45 in Rad53 recruitment to stalled forks, we combined the *cdc45-2A* mutant with the *mrc1-AQ* mutant, which lacks all 17 Mec1 phosphorylation sites and cannot bind to Rad53 (Osborn and Elledge, 2003). Importantly, combining *cdc45-2A* with *mrc1-AQ* resulted in a synergistic loss of viability in the presence of fork-stalling agents (Figure 6A), which was not due to a defect in Rad53 activation (Figure 6B). Another factor that has been suggested to recruit Rad53 to stalled forks is the DNA repair helicase Sgs1 (Hegnauer et al., 2012), but we did not detect any exacerbation of the defect of the *mrc1-AQ* and *cdc45-2A* mutants with the *sgs1-r1* mutant that cannot bind to Rad53 (Figure S6D; Hegnauer et al., 2012).

Since both Cdc45-2A and Mrc1 mutants affect origin-firing dynamics in the presence of fork-stalling agents (Figure S4A; Alcasabas et al., 2001), we wanted to rule out any role for origin firing differences on this synthetic lethality. To this end, the growth assay in Figure 6A was performed in the presence of the *sld3-A* and *dbf4-A* alleles, which allow initiation at almost all origins in HU (Figure S4C). As the *cdc45-2A* mutant showed synthetic growth defects with *mrc1-AQ* in HU with or without *sld3-A* and *dbf4-A* (Figures 6A and S6E), we conclude that recruitment of Rad53 by both Cdc45 and Mrc1 is important for cells to survive fork-stalling events independently of the roles of these proteins in regulating origin firing.

To analyze the importance of both Mrc1 and Cdc45 in recruiting Rad53 to the replisome, we performed a coIP with Mcm4, as in Figure 5D. We observed that the *cdc45-2A* mutation resulted in reduced interaction with the replisome when combined with the *mrc1-AQ* mutant (Figure S6F), again showing that Cdc45 is important to recruit Rad53 to forks. Despite this, there was still residual Rad53 interaction with the replisome, even in the *cdc45-2A mrc1-AQ* double mutant, confirming that there are yet additional mechanisms that recruit Rad53 to the replisome (see Discussion).

Given that there are multiple interactions between the replisome and Rad53, we set out to test the importance of Cdc45 recruitment of Rad53 to stalled forks in a different way. Hyperactivation of Rad53, through mutations in the phosphatases Ptc2 and Pph3 that are required to turn off Rad53, leads to inhibition of fork progression (Szyjka et al., 2008). As a result, these phosphatase mutants are sick on low doses of fork-stalling agents (Figure 6C and Szyjka et al., 2008). We reasoned that if Cdc45 recruitment of Rad53 is important for regulating fork progression, then the Cdc45-2A mutant that fails to interact with Rad53 might be able to suppress the effects of hyper-active Rad53. Importantly, combination of *cdc45-2A* with null mutations in *PTC2* and *PPH3* indeed enhanced growth in low doses of fork-stalling agents (Figure 6C), which was not due to a defect in Rad53 activation (Figure 6D). Significantly, *cdc45-2A* also suppressed the cell-cycle defect of the *ptc2Δ pph3Δ* mutants (Figure S7A), consistent with improved fork progression when hyperactive Rad53 cannot be recruited by Cdc45. Neither growth suppression nor improved cell-cycle progression can be due to defects of *cdc45-2A* in the inhibition of Sld3, as combination of *ptc2Δ pph3Δ* with the *sld3-A dbf4-A* mutants had no effect on the cell cycle or growth (Figures S7A and S7B). Together, these data show that while there are multiple interactions between Rad53 and the replisome, Cdc45 recruitment of Rad53 to stalled forks is physiologically important.

### A Meier-Gorlin Cdc45 Mutation Fails to Interact with Rad53

**Figure 7:**
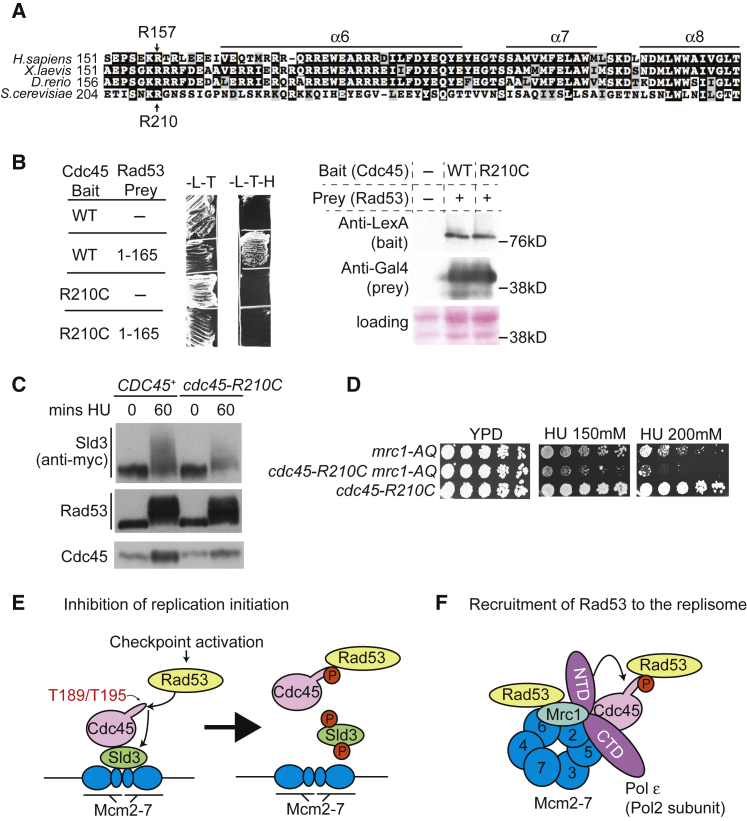
A Meier-Gorlin Cdc45 Mutation Fails to Interact with Rad53 (A) Alignment of eukaryotic Cdc45 encompassing the end of the flexible acidic loop and alpha helices 6–8. (B) Yeast-two-hybrid growth assay (left) between Cdc45 and Rad53 FHA1 (1–165) on nonselective (-L-T) and selective medium (-L-T-H). WT and R210C correspond to full-length wild-type Cdc45 and Cdc45 mutated at arginine 210 to cysteine, respectively. Right: western blot of yeast-two-hybrid fusion proteins as indicated. (C) Western blots of the indicated yeast strains, released from alpha factor (time 0) into 200 mM HU for 60 min. (D) Growth assay of the indicated strains. (E) Model of the role of Cdc45 in inhibition of origin firing. Initial Rad53-dependent phosphorylation of T189 and T195 in the acidic loop region of Cdc45 results in Rad53 binding and subsequent phosphorylation of Sld3. Rad53 phosphorylation of Sld3 inhibits its interactions with Cdc45 and Mcm2–7, as well as Dpb11 (not shown). (F) Model of the role of Cdc45 in recruiting Rad53 to the replisome. Cdc45 and Mrc1 both bind to Rad53 and both interact with Mcm2–7 subunits and the leading-strand polymerase (Pol ε). The catalytic subunit of Pol ε (Pol2) consists of a flexible N-terminal catalytic domain (NTD) and a C-terminal domain (CTD). One of the conformations of the Pol2 NTD involves an interaction with the region of Cdc45 that abuts the Rad53-binding site (arrow). DNA and many replisome components are omitted for simplicity.

Meier-Gorlin syndrome (MGS) is a rare human disease associated with multiple developmental defects and microcephaly (Kerzendorfer et al., 2013). Mutations in several replication initiation factors have been found to be causative for this disease, and recessive mutations in Cdc45 can cause many of the features of MGS (Fenwick et al., 2016). Here, we have identified a function for Cdc45 in checkpoint kinase recruitment to replication complexes, and we wondered if loss of this function might be relevant in disease. Interestingly, at least two of the MGS mutations identified in human Cdc45 occur in the unstructured loop region of this protein, which we have identified as being critical for binding to the checkpoint kinase Rad53 (Figure 2A). One patient allele exhibited complete loss of exon 5, which results in a truncated protein (I115-E162 deletion) lacking the unstructured loop, while a second allele encoded an arginine to cysteine mutation at codon 157 (Fenwick et al., 2016). Interestingly, we could identify the orthologous arginine residue in yeast Cdc45 (R210; Figure 7A) and therefore wondered if mutation of this residue to cysteine might affect the checkpoint functions of Cdc45 in yeast.

As with the *cdc45-2A* mutant, the *cdc45-R210C* mutant showed normal S phase progression and exhibited no growth defects (Figures S1A and 7D), suggesting that the essential functions of Cdc45 are not affected by this mutation. Importantly, however, this mutant showed reduced interaction with the Rad53 FHA1 domain (Figure 7B). In accordance with this, the *cdc45-R210C* allele affected Sld3 phosphorylation in HU (Figure 7C) and exhibited synthetic lethality with *mrc1-AQ* (Figure 7D). Therefore, the R210C mutation at least partially phenocopies *cdc45-2A*. These results show that mutations equivalent to those found in patients with features of MGS can cause defects in checkpoint-kinase interaction and function.

## Discussion

### Targeting of Rad53 to Replication Complexes by Cdc45 Provides Specificity

Checkpoint kinases play a crucial role in regulating multiple processes after DNA damage and fork stalling, yet how these kinases coordinate these responses is poorly understood. A significant problem in identifying bona fide checkpoint targets is that the effector kinases such as Chk1 and Rad53 have very low substrate specificities (Blasius et al., 2011, Mok et al., 2010, Sidorova and Breeden, 2003). Indeed, in an unbiased analysis of the substrate preferences of half of all yeast kinases, Rad53 was ranked as the second least specific (Mok et al., 2010), and this kinase phosphorylates at least 38 sites in Sld3, with no obvious consensus (Lopez-Mosqueda et al., 2010, Zegerman and Diffley, 2010). Despite this, from an *in vivo* screen for replication initiation targets of Rad53, we identified only two hits, Sld3 and Dbf4 (Zegerman and Diffley, 2010), suggesting that this kinase is indeed specific for substrates *in vivo*.

In this study, we show that Cdc45 acts as targeting factor for Rad53, providing specificity for Sld3 phosphorylation *in vivo*. Once active Rad53 is in proximity with Sld3, the low phosphorylation selectivity of this kinase leads to highly efficient phosphorylation of this target and multiple layers of inhibition (Deegan et al., 2016, Lopez-Mosqueda et al., 2010, Zegerman and Diffley, 2010). For Dbf4, another substrate of Rad53, specificity is likely ensured by a direct interaction between Rad53 and the N terminus of Dbf4 (Chen et al., 2013, Matthews et al., 2014), and an interaction between Rad53 and Cdc7, the enzymatic partner of Dbf4, has also been demonstrated (Aucher et al., 2010). Therefore, a comprehensive understanding of how the checkpoint kinases are targeted to different complexes might be key for identifying new substrates *in vivo*.

We have also shown that Rad53 can phosphorylate Cdc45 *in vitro* and *in vivo* (Figure 4). As phosphorylation of T189 or T195 is sufficient to create a Rad53-FHA docking site (Figure 2D), our data suggest that Rad53 phosphorylation of T189 or T195 generates its own binding site on Cdc45 (Figure 7E). This mechanism may ensure that only active Rad53 can be recruited to Cdc45 during replication. In addition, phosphorylation of Sld3 results in inhibition of the interaction with Cdc45 (Zegerman and Diffley, 2010), suggesting that this reaction becomes self-limiting (Figure 7E). A self-limiting reaction may be important to ensure that Sld3 can be rapidly de-phosphorylated and reactivated when the checkpoint is turned off. How Rad53 specifically targets Cdc45 in the first place is not yet known.

### Role of the Rad53-Cdc45 Interaction in the Replisome

Using both ChIP and IPs, we show that Rad53 interacts with the replisome during fork stalling (Figure 5). This recruitment of Rad53 is in part Cdc45 dependent, and the interaction between Rad53 and Cdc45 is physiologically important, particularly in cells that also lack the Mrc1-Rad53 interaction (Figure 6A). The synergistic lethality of the Mrc1 and Cdc45 Rad53-binding mutants suggests that the recruitment of Rad53 by these proteins is in some way redundant and may result in the phosphorylation of an overlapping set of targets. In addition to Cdc45 and Mrc1, there are also other interactions between Rad53 and the replisome (Figure S6F). Previous studies have shown that Rad53 can bind to the RecQ helicase Sgs1, which interacts with the replication fork (Cobb et al., 2003, Hegnauer et al., 2012). Despite this, a mutant of Sgs1 (*sgs-r1*), which can no longer bind to Rad53 (Hegnauer et al., 2012), did not exacerbate the lethality of the *mrc1-AQ* or *cdc45-2A* mutants in HU (Figure S6D). This does not exclude a role for Sgs1 recruitment of Rad53 in other DNA-damage contexts.

Even though *mrc1-AQ* and *cdc45-2A* mutants affect Rad53 binding at the fork, Rad53 is still activated relatively normally in these strains (Figure 6B). Therefore, there must be additional pathways that ensure that Rad53 is activated at stalled forks. An understanding of the full set of interactions of Rad53 with the replisome and the consequence of these interactions on replisome stability will be an important next step to understand Rad53 function and activation at the fork.

Intriguingly, Mrc1 and Cdc45 not only both bind to Rad53 but also interact with adjacent subunits of the Mcm2–7 helicase (Mcm6 and Mcm2, respectively; Komata et al., 2009, Sun et al., 2015). In addition, Cdc45 and Mrc1 exhibit dynamic interactions with Pol2, the catalytic subunit of the leading-strand polymerase, Pol ε (Lou et al., 2008, Sun et al., 2015, Zhou et al., 2017). Pol2 consists of a flexible N-terminal catalytic domain and a C-terminal non-catalytic domain (Figure 7F). Mrc1 binds to both domains of Pol2, but the interaction with the Pol2 N terminus appears to be lost after fork stalling (Lou et al., 2008). Cdc45 also binds to the Pol2 N terminus (Sun et al., 2015, Zhou et al., 2017), and interestingly, this interaction appears to be through the alpha helix 6 of Cdc45, which is adjacent to the loop region that binds to Rad53 (Figures 7A and S2D). Given the interactions of Mrc1 and Cdc45 with Rad53, Pol2 and adjacent subunits of Mcm2–7 (Komata et al., 2009, Sun et al., 2015), it will be important now to understand the interplay between these interactions in the context of signaling that the fork has stalled and ensuring that the fork resumes DNA synthesis after stalling (Figure 7F).

### Conservation of Replication-Checkpoint Interactions in Humans

There are significant similarities in the checkpoint-dependent regulation of origin firing between yeast and metazoa, including inhibition of the Sld3 ortholog Treslin (Boos et al., 2011), inhibition of DDK (Costanzo et al., 2003, Lee et al., 2012), and release of Cdc45 from chromatin (Costanzo et al., 2000, Liu et al., 2006). Despite this, the checkpoint-dependent mechanisms that regulate these pathways in metazoa are poorly understood. Chk1 binds to Treslin, but this interaction was shown to be involved in the regulation of normal S phase progression, not for the inhibition of origin firing after fork stalling (Guo et al., 2015). Given the low sequence specificity of the checkpoint effector kinases (Blasius et al., 2011, Mok et al., 2010), we expect that the targeting of these kinases will be a critical determinant of the response to replication stress across organisms.

Currently, very little is known about how the checkpoint kinases interact with stalled replisomes after replication stress in metazoa. Chk1 has been shown to interact with CMG complex components in mammalian cells (Han et al., 2014), and Chk2 can phosphorylate and regulate the *Drosophila* CMG complex *in vitro* (Ilves et al., 2012). From the work presented here, it is possible that the unstructured loop of Cdc45 that protrudes away from the Mcm2–7 and GINS interfaces in the human CMG complex (Simon et al., 2016) may target the checkpoint kinases to the metazoan replisome.

We show that a yeast *CDC45* mutation that is orthologous to a mutation in a patient with features of MGS fails to interact with the checkpoint kinase Rad53 (Figure 7B). Although it remains to be seen whether human Cdc45 has similar interactions with checkpoint kinases, it is intriguing that checkpoint mutations also cause human diseases, such as Seckel syndrome, that share overlapping features with MGS, including microcephaly (Kerzendorfer et al., 2013). By understanding the interactions between DNA replication and the checkpoint response, it may be possible to derive mechanistic insights into the clinical phenotypes of patients who harbor mutations in these pathways. Furthermore, as replication stress and checkpoint activation are early events in tumor progression and consequences of many chemotherapies (Lecona and Fernández-Capetillo, 2014, Macheret and Halazonetis, 2015), understanding how the checkpoint regulates DNA replication will have implications for cancer therapy.

## STAR★Methods

### Key Resources Table

**Table:** 

REAGENT or RESOURCE	SOURCE	IDENTIFIER
**Antibodies**		

Polyclonal rabbit anti-Rad53	Abcam	Cat no: ab104232; RRID: AB_2687603
Polyclonal rabbit anti-Cdc45	This paper	N/A
Monoclonal mouse anto-Myc(9E10)	Roche	Cat no: 11 667 149 001; RRID: AB_390912
Monoclonal mouse anti-HA (16B12)	Abcam	Cat no: ab130275; RRID: AB_11156884

**Bacterial Strains**		

BL21 RIL	Agilent Technologies	Cat No: 230240
Yeast Strains	See Table S1	

**Chemicals, Peptides, and Recombinant Proteins**		

PhosTag Acrylamide	Wako Chemicals	Cat no: 304-93521
EGS (ethylene glycol bis(succinimidyl succinate))	Thermo Fisher	Cat no: 21565
DSP (dithiobis(succinimidyl propionate))	Thermo Fisher	Cat no: 22585

**Deposited Data**		

Raw and analyzed data. GEO submission.	This paper	GSE122110

**Recombinant DNA**		

pET30Z Cdc45 1-238	This paper	bPZ78
pET21b Rad53	Zegerman and Diffley, 2010	bPZ192
pET30a Sld3 FL	Zegerman and Diffley, 2010	bPZ13
pET21b Cdc45 7HIS 444-450	This paper	bPZ941
pET21b Cdc45 2A 7HIS 444-450	This paper	bPZ967
pET30a Sld3 530-668	Zegerman and Diffley, 2010	bPZ367

**Software and Algorithms**		

Sickel (Version 1.33)		https://github.com/najoshi/sickle
Trim Galore (Version 0.4.2)		https://github.com/FelixKrueger/TrimGalore
Bowtie2 (Version 2.2.6)		http://bowtie-bio.sourceforge.net/index.shtml
DeepTools (Version 3.1.2)		https://deeptools.readthedocs.io/en/develop/
BLAT (version 35)		https://users.soe.ucsc.edu/∼kent/src/
SeqPlot (3.0.12)		https://github.com/Przemol/seqplots

**Critical Commercial Assays**		

TruSeq Nano DNA LT Sample Prep Kit	Illumina	Cat no: FC-121-4002
LightCycler 480 SYBR green 1 master mix	Roche	Cat no: 04707516001

### Contact for Reagent and Resource Sharing

Further information and requests for resources and reagents should be directed to and will be fulfilled by the Lead Contact, Philip Zegerman (paz20@cam.ac.uk).

### Experimental Model and Subject Details

Yeast strains are listed in the Key Resources Table.

### Method Details

Unless otherwise stated, the data in the figures are representative of 3 biological replicates.

#### *In vitro* kinase assays

Rad53-6HIS and Sld3-6HIS were purified using Ni-NTA chromatography. In budding yeast, C-terminal and N-terminal tagged Cdc45 generates hypomorphic CDC45 mutants. Therefore we decided to internally tag Cdc45. For this, amino acids in the poorly conserved loop region 444-450 were mutated to histidine to generate Cdc45-(444-450)-7HIS, hereafter called Cdc45-7HIS. By replacement of endogenous *CDC45* in yeast, we checked that Cdc45-7HIS had normal Rad53 activation, normal phosphorylation of Sld3 and was not synthetic lethal with *rad53Δ* mutants. We are confident therefore that this internally tagged Cdc45 behaves as wild-type.

Cdc45-7HIS was expressed from pET21b in BL21 RIL and purified by affinity using His-HP column (GE healthcare) in buffer A (20mM HEPES pH8.2, 300mM NaCl, 5% glycerol, 20mM Imidazole pH8 + protease inhibitors). Eluted protein was exchanged into buffer R (10mM HEPES pH7.2, 150mM NaCl, 0.005% Tween 20, 5% glycerol) +3mM EDTA pH 8. For the kinase assay Rad53 (0.18pmole), Sld3 (4.5pmole) with BSA (4.5pmole) or Cdc45 (4.5pmole) were preincubated on ice for 30mins in buffer R. To start the reaction an equal volume of buffer R’ (20mM HEPES pH7.2, 20mM MgCl_2_, 200 μM ATP +γATP) was added and the reaction was placed at 37°C. Reactions were stopped by addition of Laemmli buffer and freezing before resolution by SDS-PAGE and autoradiography. The Cdc45-2A mutant was tagged and purified exactly as for the wild-type protein.

##### Peptide pulldown experiments

Peptides corresponding to Cdc45 185-203, with or without phospho-threonines at position 189 and 195, were synthesized by Genscript with a C-terminal cysteine residue. Lyophilised peptides were solubilised in water and diluted to 0.1mg/ml in coupling buffer (50mM Tris pH 8.5, 5mM EDTA). 0.5ml of diluted peptide was added to 0.5ml of Sulfolink beads (Pierce) and incubated for 60 minutes with rotation at room temperature. Remaining active groups on the resin were quenched by addition of 50mM cysteine for 45 minutes at room temperature. Beads were washed 2 times in 1ml 1M NaCl, then washed into pulldown buffer (20mM Tris pH8, 5% glycerol, 300mM NaCl) and stored at 4°C. GST (pGEX 2TKP) or Rad53-(1-165)-GST (in pET21b) were expressed in BL21 RP bacteria by overnight incubation in 2TY+0.5mM IPTG at 16°C and pellets were frozen. For the pulldown, extracts were made from 25ml of starting bacterial culture pellet and sonicated in pulldown buffer + protease inhibitors (leupeptin, pepstatin, benzamidine HCl and PMSF) + PhosStop (Roche, 1 tablet per 100ml). After centrifugation, supernatants were used in pulldowns as follows; 50 μL sulfolink coupled peptides of beads + 200 μL pulldown buffer + 50 μL extract and incubated for 45 minutes at 4°C with rotation. Pulldowns were washed 3 times in pulldown buffer and resolved on 15% SDS-PAGE gel followed by Coomassie staining.

#### Yeast methods, western blotting and antibodies

Polyclonal rabbit anti-Rad53 was from Abcam (ab104232), used 1 in 5000 in TBST (TBS + 0.1% Tween 20) + 5% milk powder. Polyclonal rabbit Cdc45 antibodies were generated as follows. Cdc45 (1-238)-6His was expressed from pET30Z in BL21 DE3 pLysS and purified by affinity using His-HP column (GE healthcare) in lysis buffer (8M Urea, 100mM NaPhosphate buffer pH7, 250mM NaCl, 20mM Imidazole pH 8.0, 10% glycerol, 0.5% Triton X-100). Ammonium sulfate precipitated protein at 4mg/ml was used as immunogen (Biogenes). For affinity purification, Cdc45 (1-238) fragment was coupled to CNBr Sepharose beads according to the manufacturers instructions (CNBr-Activated Sepharose 4 Fast Flow - GE Healthcare) in coupling buffer (4M Urea, 0.2 M NaCO3, 1M NaCl at pH9). Serum was incubated with Cdc45 (1-238)-CNBR beads and washed three times with PBS pH 8.0. Antibodies were eluted with 200mM Glycine pH 2.8 on ice and equilibrated immediately with 1M Tris pH 8.0. Affinity purified Cdc45 was used at 1 in 500 in TBST + 5% milk powder.

#### Southern blot of replication intermediates on alkali gels

Yeast strains were arrested at 25°C in alpha factor and released into 200mM HU. DNA was isolated using lyticase treatment in lysis buffer (2% Triton X-100, 1% SDS, 100mM NaCl, 10mM Tris-HCl pH7.8, 1mM EDTA, 1% β-mercaptoethanol), followed by phenol chloroform extraction. After RNase A treatment, the DNA was run on 1% agarose gel (50mM NaOH, 1mM EDTA) for 17 hours at 25V. DNA was visualized by Southern blotting to hybond XL and hybridization to radioactive ARS probes made using Prime-A-Gene (Promega).

#### Phostag SDS-PAGE

Agarose supported SDS-polyacrylamide gels containing phostag reagent were used to resolve phosphorylated Mrc1-13myc and Cdc45. The resolving gels were 0.5% agarose, 375 mM Tris pH 8.8, 0.1% SDS, 0.001% TEMED, 0.035 mM MnCl2, 0.05% APS, 0.0125 mM Phos-tag acrylamide (Alpha Laboratories Ltd) and either 3% acrylamide:bisacrylamide (29:1) for Mrc1 or 5% for Cdc45. The stacking gels were composed of 3% acrylamide:bisacrylamide (29:1), 125 mM Tris pH 6.8, 0.001% TEMED, 0.05% APS. Gels were washed three times for 15 minutes with 50mM EDTA and once for 15 minutes with western blot transfer buffer before blotting.

#### ChIP-seq

200 mL of yeast culture was crosslinked with 1.5mM EGS (ethylene glycol bis(succinimidyl succinate)) for 10 minutes, and then Formaldehyde was added at final concentration of 1% for 10 additional minutes at room temperature with gentle rotation. Crosslinking reactions were terminated by addition of 125 mM Glycine for 20 minutes at room temperature with gentle rotation. Cells were washed 3 times with PBS, once with 50 mM HEPES and resuspended in lysis buffer (50mM HEPES/KOH pH7.5, 1mM EDTA, 1% Triton X-100, 0.1% Sodium deoxycholate, 140mM NaCl, Phosphatase Inhibitor Cocktail 2 and 3 (Sigma), Protease inhibitors (Roche)). 300 μL of glass beads were added and cells were mechanically disrupted with tissue homogenizer at 4°C (Precellys) for 10 cycles at 6000 rpm for 30 s, with 3 minutes incubation on ice between each cycle. Cell lysates were sonicated 25 cycles of 30 s on and 30 s off and insoluble material was discarded by centrifugation at 13000rpm for 20 minutes at 4°C. Supernatants were transferred to new tubes and anti-HA conjugated magnetic beads were added to the cell lysates. Beads were incubated with samples overnight at 4°C. Beads were collected and washed twice with lysis buffer, once with buffer 1 (50mM HEPES/KOH pH7.5, 1mM EDTA, 1% Triton X-100, 0.1% Sodium deoxycholate and 250mM NaCl), once with buffer 2 (50mM HEPES/KOH pH7.5, 1mM EDTA, 1% Triton X-100, 0.1% Sodium deoxycholate and 500mM NaCl), once with buffer 3 (0.25M LiCl, 0.5% NP-40, 0.5% Sodium deoxycholate, 1mM EDTA, 10mM Tris-HCl pH8) and twice with TE pH8. Samples were eluted in elution buffer (0.85X TE pH8, 1% SDS, 0.25M NaCl) for 30 minutes at 65°C. Eluted materials were transferred to new tubes and treated with RNase A for 1 hour at 37°C. Then samples were treated with Proteinase K overnight at 65°C. DNAs were purified with phenol/chloroform extraction. DNaseq libraries were prepared according to instructions of Illumina truseq-Nano except adaptors were used at 1 to 100 dilution. Sequencing was performed using an Illumina Hiseq 1500.

##### Mcm4-HA Immunoprecipitation (IP)

200 mL yeast cultures were crosslinked with1.5mM EGS or DSP (dithiobis(succinimidyl propionate)) for 15 minutes and the reactions were quenched by addition of 125 mM Glycine for 20 minutes at room temperature with gentle rotation. Cells were washed 3 times with PBS and resuspended in Lysis buffer (50mM HEPES/KOH pH7.5, 1mM EDTA, 140mM NaCl, 1mM EDTA, Phosphatase and Protease inhibitors). 300 μL of glass beads were added and cells were mechanically disrupted with tissue homogenizer at 4°C (Precellys) for 10 cycles at 6000 rpm for 30 s, with 3 minutes incubation on ice between each cycle. Cell lysates were transferred into new tubes and sonicated 3 cycles of 30 s on and 30 s off. 1500 units of nuclease (Pierce universal nuclease) was added to samples and incubated on a rotator for 2 hours at 4°C. 0.25 volume of extraction buffer (50 mM HEPES-KOH pH 7.5, 140 mM, 1 mM EDTA, 5% Triton X-100, 0.5% Sodium deoxycholate) was added and incubated on a rotator for an additional 30 minutes at 4°C. Insoluble material was discarded by centrifugation at 13000rpm for 10 minutes at 4°C. Supernatants were transferred to new tubes and anti-HA conjugated magnetic beads were added to the cell lysates. Beads were incubated with samples overnight at 4°C and washed three times each with buffer 1 (50 mM HEPES-KOH pH 7.5, 500 mM NaCl, 1% Triton X-100, 0.1% sodium deoxycholate, 1 mM EDTA) and buffer 2 (10 mM Tris-HCl pH 8.0, 250 mM LiCl, 0.5% NP-40, 0.5% sodium deoxycholate, 1 mM EDTA). To cleave the EGS crosslinks 0.5M hydroxlamine pH8.5 (dissolved in PBS) was added to beads and incubated for 30 minutes at 37°C. Proteins were eluted from beads by addition of 2X Laemmli buffer and incubation 15 minutes at 95°C. To cleave the DSP crosslinks samples were incubated for 15 minutes at 95°C in modified Laemmli buffer (67.5 mM Tris-HCl pH 6.8, 2% SDS, 10% Glycerol, 100 mM DTT, 0.01% Bromophenol Blue).

### Quantification and Statistical Analysis

#### Bioinformatic analysis ChIP-seq

FastQ files were filtered for low quality reads (< Q20) and low quality bases were trimmed from the ends of the reads (< Q20) using Sickle (Version 1.33). Adapters were removed using Trim Galore (version 0.4.2). The resulting reads were mapped against the W303 genome (GeneBank: LYZE00000000) using Bowtie2 (2.2.6). Origin sequences and timings were retrieved from OriDB (Siow et al., 2012) and centromeric sequences were retrieved from The *Saccharomyces* Genome Database. These features were annotated against the W303 genome by sequence matching with BLAT (version 35).

A dormant replicative region was defined as the 10 kb region between two late/dormant origins of maximal base pair distance. It was assumed that there was no replication in such regions after 1h HU treatment. 10 such origin pairs were chosen as the dormant region set. Replication profiles were obtained using the following methodology:

1) S-phase samples were normalized to G1 samples by binning each chromosomal region to 100bps and dividing the G1 sample counts over the S-phase samples counts.
2) The average count of the dormant region set from all samples was taken as 1N.
3) The relative count of all regions in 100bp bins was calculated as a ratio of 1N.
4) Relative ratios bigger than 2.2N were masked out.

ChIP data was analyzed by normalizing the read counts to ± 500bp of centromeric reads. Normalization and processing were done using DeepTools (v3.1.2). For visualization, centromeric regions were masked out. All graphs were produced by SeqPlot.

### Data and Software Availability

Sequencing data is available at GEO: GSE122110.

## Supplemental Information

Document S1. Figures S1–S7

Table S1. Yeast Strains, Related to STAR Methods

Document S2. Article plus Supplemental Information

## References

[bib1] Alcasabas A.A., Osborn A.J., Bachant J., Hu F., Werler P.J., Bousset K., Furuya K., Diffley J.F., Carr A.M., Elledge S.J. (2001). Mrc1 transduces signals of DNA replication stress to activate Rad53. Nat. Cell Biol..

[bib2] Aucher W., Becker E., Ma E., Miron S., Martel A., Ochsenbein F., Marsolier-Kergoat M.C., Guerois R. (2010). A strategy for interaction site prediction between phospho-binding modules and their partners identified from proteomic data. Mol. Cell. Proteomics.

[bib3] Bell S.P., Labib K. (2016). Chromosome duplication in *Saccharomyces cerevisiae*. Genetics.

[bib4] Blasius M., Forment J.V., Thakkar N., Wagner S.A., Choudhary C., Jackson S.P. (2011). A phospho-proteomic screen identifies substrates of the checkpoint kinase Chk1. Genome Biol..

[bib5] Boos D., Sanchez-Pulido L., Rappas M., Pearl L.H., Oliver A.W., Ponting C.P., Diffley J.F. (2011). Regulation of DNA replication through Sld3-Dpb11 interaction is conserved from yeast to humans. Curr. Biol..

[bib6] Chen S.H., Zhou H. (2009). Reconstitution of Rad53 activation by Mec1 through adaptor protein Mrc1. J. Biol. Chem..

[bib7] Chen Y.C., Kenworthy J., Gabrielse C., Hänni C., Zegerman P., Weinreich M. (2013). DNA replication checkpoint signaling depends on a Rad53-Dbf4 N-terminal interaction in *Saccharomyces cerevisiae*. Genetics.

[bib8] Cobb J.A., Bjergbaek L., Shimada K., Frei C., Gasser S.M. (2003). DNA polymerase stabilization at stalled replication forks requires Mec1 and the RecQ helicase Sgs1. EMBO J..

[bib9] Conde F., Ontoso D., Acosta I., Gallego-Sánchez A., Bueno A., San-Segundo P.A. (2010). Regulation of tolerance to DNA alkylating damage by Dot1 and Rad53 in *Saccharomyces cerevisiae*. DNA Repair (Amst.).

[bib10] Costanzo V., Robertson K., Ying C.Y., Kim E., Avvedimento E., Gottesman M., Grieco D., Gautier J. (2000). Reconstitution of an ATM-dependent checkpoint that inhibits chromosomal DNA replication following DNA damage. Mol. Cell.

[bib11] Costanzo V., Shechter D., Lupardus P.J., Cimprich K.A., Gottesman M., Gautier J. (2003). An ATR- and Cdc7-dependent DNA damage checkpoint that inhibits initiation of DNA replication. Mol. Cell.

[bib12] De Piccoli G., Katou Y., Itoh T., Nakato R., Shirahige K., Labib K. (2012). Replisome stability at defective DNA replication forks is independent of S phase checkpoint kinases. Mol. Cell.

[bib13] Deegan T.D., Yeeles J.T., Diffley J.F. (2016). Phosphopeptide binding by Sld3 links Dbf4-dependent kinase to MCM replicative helicase activation. EMBO J..

[bib14] Dungrawala H., Rose K.L., Bhat K.P., Mohni K.N., Glick G.G., Couch F.B., Cortez D. (2015). The replication checkpoint prevents two types of fork collapse without regulating replisome stability. Mol. Cell.

[bib15] Errico A., Costanzo V. (2012). Mechanisms of replication fork protection: a safeguard for genome stability. Crit. Rev. Biochem. Mol. Biol..

[bib16] Fenwick A.L., Kliszczak M., Cooper F., Murray J., Sanchez-Pulido L., Twigg S.R., Goriely A., McGowan S.J., Miller K.A., Taylor I.B., WGS500 Consortium (2016). Mutations in CDC45, encoding an essential component of the pre-initiation complex, cause Meier-Gorlin syndrome and craniosynostosis. Am. J. Hum. Genet..

[bib17] Gan H., Yu C., Devbhandari S., Sharma S., Han J., Chabes A., Remus D., Zhang Z. (2017). Checkpoint kinase Rad53 couples leading- and lagging-strand DNA synthesis under replication stress. Mol. Cell.

[bib18] Giannattasio M., Branzei D. (2017). S-phase checkpoint regulations that preserve replication and chromosome integrity upon dNTP depletion. Cell. Mol. Life Sci..

[bib19] Guo C., Kumagai A., Schlacher K., Shevchenko A., Shevchenko A., Dunphy W.G. (2015). Interaction of Chk1 with Treslin negatively regulates the initiation of chromosomal DNA replication. Mol. Cell.

[bib20] Han X., Aslanian A., Fu K., Tsuji T., Zhang Y. (2014). The interaction between checkpoint kinase 1 (Chk1) and the minichromosome maintenance (MCM) complex is required for DNA damage-induced Chk1 phosphorylation. J. Biol. Chem..

[bib21] Hegnauer A.M., Hustedt N., Shimada K., Pike B.L., Vogel M., Amsler P., Rubin S.M., van Leeuwen F., Guénolé A., van Attikum H. (2012). An N-terminal acidic region of Sgs1 interacts with Rpa70 and recruits Rad53 kinase to stalled forks. EMBO J..

[bib22] Ilves I., Tamberg N., Botchan M.R. (2012). Checkpoint kinase 2 (Chk2) inhibits the activity of the Cdc45/MCM2-7/GINS (CMG) replicative helicase complex. Proc. Natl. Acad. Sci. USA.

[bib23] Katou Y., Kanoh Y., Bando M., Noguchi H., Tanaka H., Ashikari T., Sugimoto K., Shirahige K. (2003). S-phase checkpoint proteins Tof1 and Mrc1 form a stable replication-pausing complex. Nature.

[bib24] Kerzendorfer C., Colnaghi R., Abramowicz I., Carpenter G., O’Driscoll M. (2013). Meier-Gorlin syndrome and Wolf-Hirschhorn syndrome: two developmental disorders highlighting the importance of efficient DNA replication for normal development and neurogenesis. DNA Repair (Amst.).

[bib25] Komata M., Bando M., Araki H., Shirahige K. (2009). The direct binding of Mrc1, a checkpoint mediator, to Mcm6, a replication helicase, is essential for the replication checkpoint against methyl methanesulfonate-induced stress. Mol. Cell. Biol..

[bib26] Labib K. (2010). How do Cdc7 and cyclin-dependent kinases trigger the initiation of chromosome replication in eukaryotic cells?. Genes Dev..

[bib27] Labib K., De Piccoli G. (2011). Surviving chromosome replication: the many roles of the S-phase checkpoint pathway. Philos. Trans. R. Soc. Lond. B Biol. Sci..

[bib28] Lecona E., Fernández-Capetillo O. (2014). Replication stress and cancer: it takes two to tango. Exp. Cell Res..

[bib29] Lee A.Y., Chiba T., Truong L.N., Cheng A.N., Do J., Cho M.J., Chen L., Wu X. (2012). Dbf4 is direct downstream target of ataxia telangiectasia mutated (ATM) and ataxia telangiectasia and Rad3-related (ATR) protein to regulate intra-S-phase checkpoint. J. Biol. Chem..

[bib30] Liu P., Barkley L.R., Day T., Bi X., Slater D.M., Alexandrow M.G., Nasheuer H.P., Vaziri C. (2006). The Chk1-mediated S-phase checkpoint targets initiation factor Cdc45 via a Cdc25A/Cdk2-independent mechanism. J. Biol. Chem..

[bib31] Lopez-Mosqueda J., Maas N.L., Jonsson Z.O., Defazio-Eli L.G., Wohlschlegel J., Toczyski D.P. (2010). Damage-induced phosphorylation of Sld3 is important to block late origin firing. Nature.

[bib32] Lou H., Komata M., Katou Y., Guan Z., Reis C.C., Budd M., Shirahige K., Campbell J.L. (2008). Mrc1 and DNA polymerase epsilon function together in linking DNA replication and the S phase checkpoint. Mol. Cell.

[bib33] Macheret M., Halazonetis T.D. (2015). DNA replication stress as a hallmark of cancer. Annu. Rev. Pathol..

[bib34] Matthews L.A., Selvaratnam R., Jones D.R., Akimoto M., McConkey B.J., Melacini G., Duncker B.P., Guarné A. (2014). A novel non-canonical forkhead-associated (FHA) domain-binding interface mediates the interaction between Rad53 and Dbf4 proteins. J. Biol. Chem..

[bib35] Mok J., Kim P.M., Lam H.Y., Piccirillo S., Zhou X., Jeschke G.R., Sheridan D.L., Parker S.A., Desai V., Jwa M. (2010). Deciphering protein kinase specificity through large-scale analysis of yeast phosphorylation site motifs. Sci. Signal..

[bib36] Osborn A.J., Elledge S.J. (2003). Mrc1 is a replication fork component whose phosphorylation in response to DNA replication stress activates Rad53. Genes Dev..

[bib37] Pardo B., Crabbé L., Pasero P. (2017). Signaling pathways of replication stress in yeast. FEMS Yeast Res..

[bib38] Raghuraman M.K., Winzeler E.A., Collingwood D., Hunt S., Wodicka L., Conway A., Lockhart D.J., Davis R.W., Brewer B.J., Fangman W.L. (2001). Replication dynamics of the yeast genome. Science.

[bib39] Remus D., Diffley J.F. (2009). Eukaryotic DNA replication control: lock and load, then fire. Curr. Opin. Cell Biol..

[bib40] Saldivar J.C., Cortez D., Cimprich K.A. (2017). The essential kinase ATR: ensuring faithful duplication of a challenging genome. Nat. Rev. Mol. Cell Biol..

[bib41] Sheu Y.J., Stillman B. (2010). The Dbf4-Cdc7 kinase promotes S phase by alleviating an inhibitory activity in Mcm4. Nature.

[bib42] Sidorova J.M., Breeden L.L. (2003). Rad53 checkpoint kinase phosphorylation site preference identified in the Swi6 protein of Saccharomyces cerevisiae. Mol. Cell. Biol..

[bib43] Simon A.C., Sannino V., Costanzo V., Pellegrini L. (2016). Structure of human Cdc45 and implications for CMG helicase function. Nat. Commun..

[bib44] Siow C.C., Nieduszynska S.R., Müller C.A., Nieduszynski C.A. (2012). OriDB, the DNA replication origin database updated and extended. Nucleic Acids Res..

[bib45] Sun J., Shi Y., Georgescu R.E., Yuan Z., Chait B.T., Li H., O’Donnell M.E. (2015). The architecture of a eukaryotic replisome. Nat. Struct. Mol. Biol..

[bib46] Szyjka S.J., Aparicio J.G., Viggiani C.J., Knott S., Xu W., Tavaré S., Aparicio O.M. (2008). Rad53 regulates replication fork restart after DNA damage in *Saccharomyces cerevisiae*. Genes Dev..

[bib47] Tanaka S., Araki H. (2013). Helicase activation and establishment of replication forks at chromosomal origins of replication. Cold Spring Harb. Perspect. Biol..

[bib48] Tanaka K., Russell P. (2004). Cds1 phosphorylation by Rad3-Rad26 kinase is mediated by forkhead-associated domain interaction with Mrc1. J. Biol. Chem..

[bib49] Tercero J.A., Longhese M.P., Diffley J.F. (2003). A central role for DNA replication forks in checkpoint activation and response. Mol. Cell.

[bib50] Zegerman P., Diffley J.F. (2009). DNA replication as a target of the DNA damage checkpoint. DNA Repair (Amst.).

[bib51] Zegerman P., Diffley J.F. (2010). Checkpoint-dependent inhibition of DNA replication initiation by Sld3 and Dbf4 phosphorylation. Nature.

[bib52] Zhou J.C., Janska A., Goswami P., Renault L., Abid Ali F., Kotecha A., Diffley J.F.X., Costa A. (2017). CMG-Pol epsilon dynamics suggests a mechanism for the establishment of leading-strand synthesis in the eukaryotic replisome. Proc. Natl. Acad. Sci. USA.

