# Supplementary material for: Helicase Subunit Cdc45 Targets the Checkpoint Kinase Rad53 to Both Replication Initiation and Elongation Complexes after Fork Stalling
Source: Mol Cell. 2019 Feb 7;73(3):562–573.e3. doi: 10.1016/j.molcel.2018.11.025 (PMC6375734; doi:10.1016/j.molcel.2018.11.025)
Supplement: Document S1. Figures S1–S7 [file mmc1.pdf]

Molecular Cell, Volume *73*

Supplemental Information

Helicase Subunit Cdc45 Targets the Checkpoint Kinase Rad53 to Both Replication Initiation and Elongation Complexes after Fork Stalling

Geylani Can, Anastasia Christine Kauerhof, Dominik Macak, and Philip Zegerman

Figure S1

A

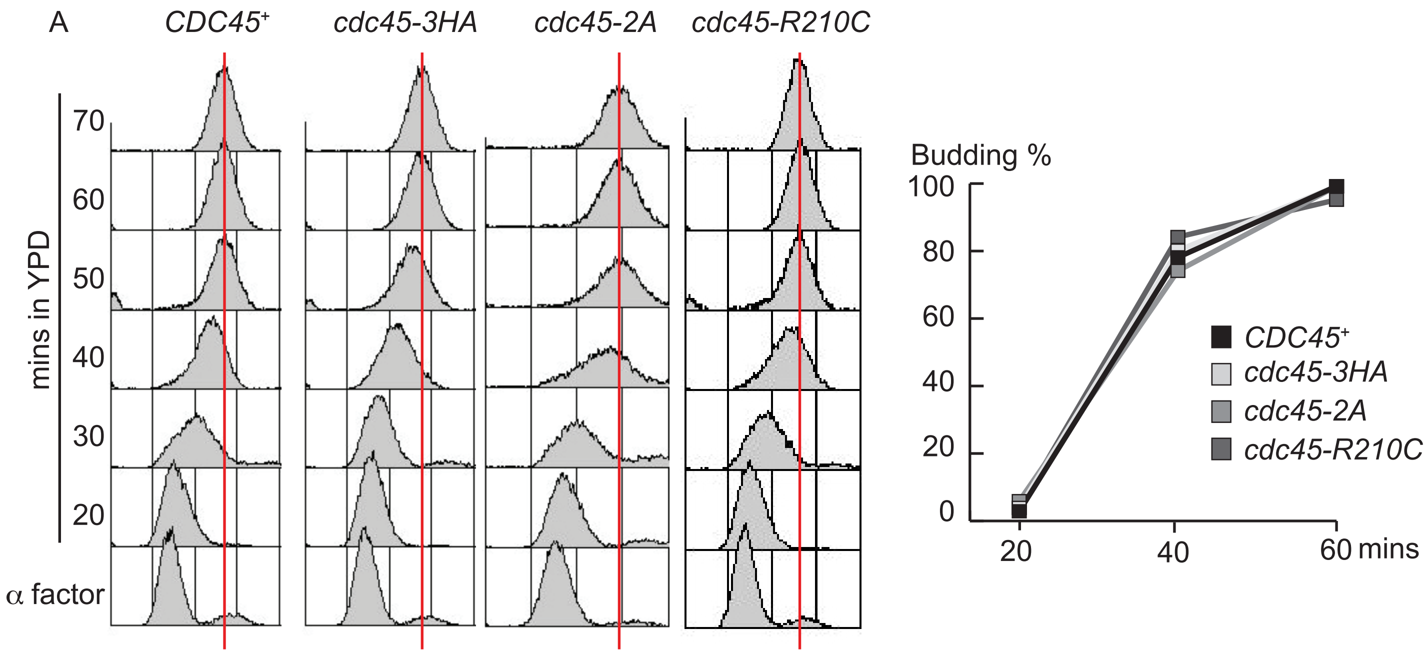

B

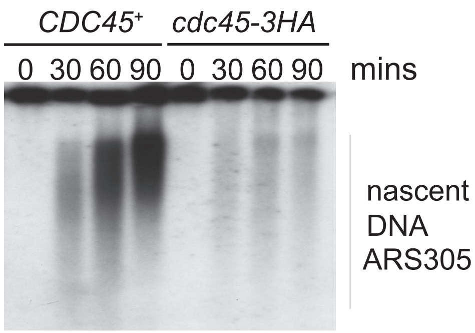

C *sml1Δ cdc45-3HA* x *sml1Δ rad53Δ*

n=64<br>genotype	expected frequency	actual frequency
sml1Δ cdc45-3HA RAD53+	25%	24%
sml1Δ cdc45-3HA rad53Δ	25%	0%

D *cdc45-2A* x *sml1Δ rad53Δ*

n=68<br>genotype	expected frequency	actual frequency
sml1Δ RAD53+ cdc45-2A	12.5%	16%
sml1Δ cdc45-2A rad53Δ	12.5%	12%

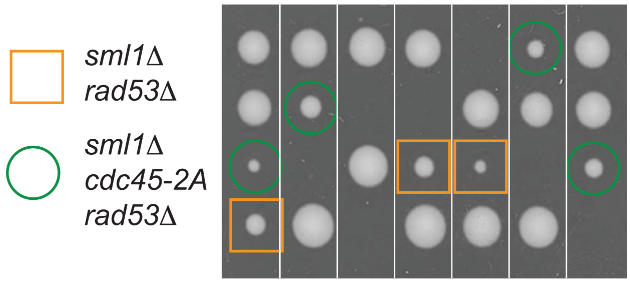

E Hydroxyurea in S-phase

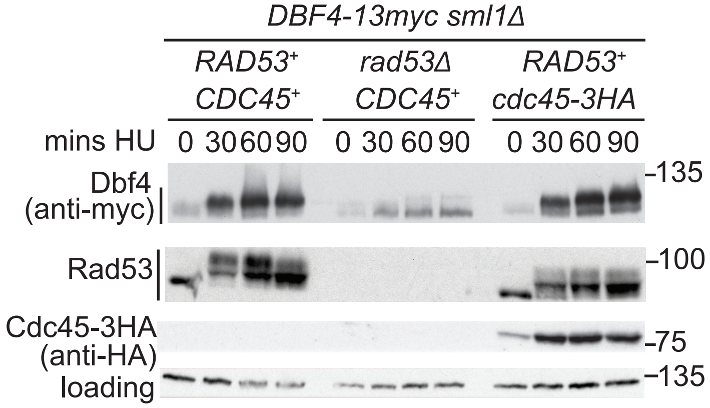

Figure S2

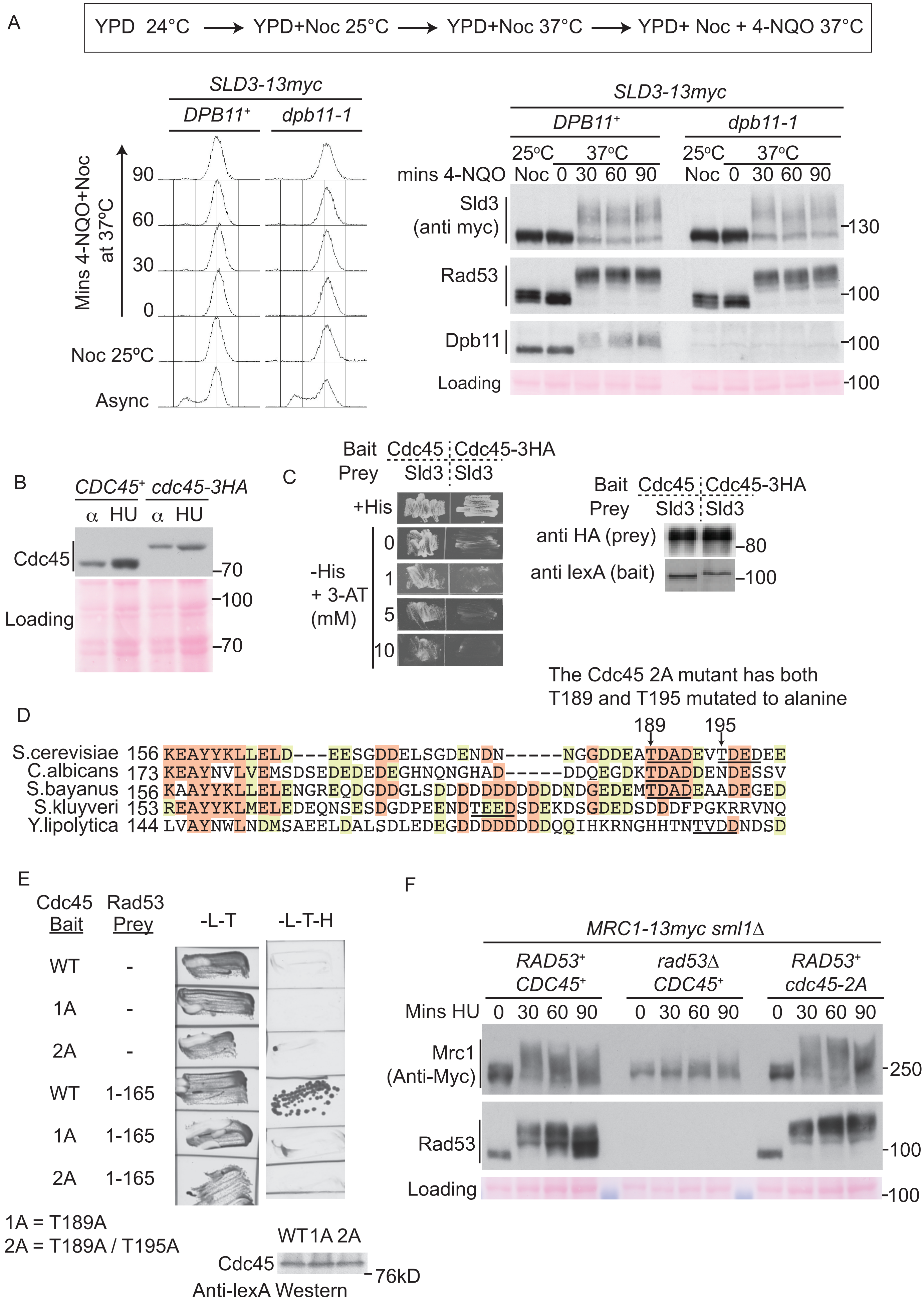
A
YPD 24°C → YPD+Noc 25°C → YPD+Noc 37°C → YPD+ Noc + 4-NQO 37°C
SLD3-13myc
DPB11+
dpb11-1
Mins 4-NQO+Noc at 37°C
90
60
30
0
Noc 25°C
Async
SLD3-13myc
DPB11+
dpb11-1
25°C
37°C
mins 4-NQO Noc 0 30 60 90
Sld3 (anti myc)
Rad53
Dpb11
Loading
130
100
B
CDC45+ cdc45-3HA
α HU α HU
Cdc45
Loading
70
100
70
C
Bait Cdc45 Cdc45-3HA
Prey Sld3 Sld3
+His
-His + 3-AT (mM)
0
1
5
10
anti HA (prey)
anti lexA (bait)
80
100
The Cdc45 2A mutant has both T189 and T195 mutated to alanine
D
189
195
S.cerevisiae 156 KEAYYKLLELD---EESGDDELSGDENDN-----NGGDDEATDADEVTDEDEE
C.albicans 173 KEAYNVLVEMSDSEDEDEDEGHNQNGHAD-----DDQEGDKTDADDENDESSV
S.bayanus 156 KAAYYKLLELENGREQDGDDGLSDDDDDDDDDNDGEDEMTDADEAADEGED
S.kluyveri 153 REAYYKLMELEDEQNSESDGDPEENDTEEDSDEKDSGDEDSDDDFPGKRRVNQ
Y.lipolytica 144 LVAYNWLNDMSAEELDALSDLEDEGDDDDDDDDQQIHKRNGHHTNTVDDNDSD
E
Cdc45 Bait
Rad53 Prey
-L-T
-L-T-H
WT -
1A -
2A -
WT 1-165
1A 1-165
2A 1-165
1A = T189A
2A = T189A / T195A
WT 1A 2A
Cdc45
76kD
Anti-lexA Western
F
MRC1-13myc sml1Δ
RAD53+ CDC45+
rad53Δ CDC45+
RAD53+ cdc45-2A
Mins HU 0 30 60 90 0 30 60 90 0 30 60 90
Mrc1 (Anti-Myc)
Rad53
Loading
250
100
100

Figure S3

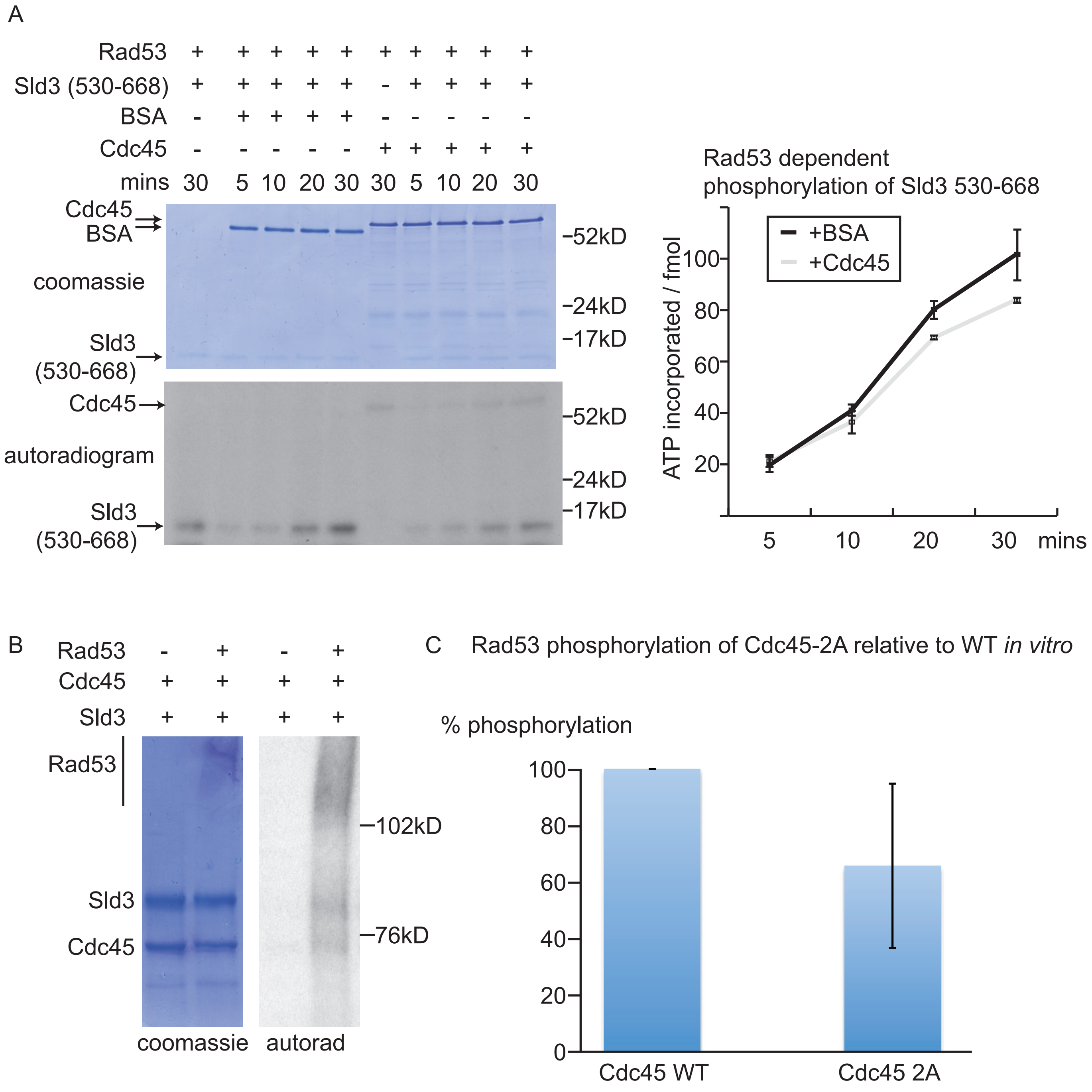
A
Rad53 + + + + + + + + + +
Sld3 (530-668) + + + + + - + + + +
BSA - + + + + - - - - -
Cdc45 - - - - - + + + + +
mins 30 5 10 20 30 30 5 10 20 30
Cdc45
BSA
coomassie
Sld3 (530-668)
-52kD
-24kD
-17kD
Cdc45
autoradiogram
Sld3 (530-668)
-52kD
-24kD
-17kD
Rad53 dependent phosphorylation of Sld3 530-668
+BSA
+Cdc45
ATP incorporated / fmol
100
80
60
40
20
5
10
20
30
mins
B
Rad53 - + - +
Cdc45 + + + +
Sld3 + + + +
Rad53
Sld3
Cdc45
-102kD
-76kD
coomassie
autorad
C Rad53 phosphorylation of Cdc45-2A relative to WT *in vitro*
% phosphorylation
100
80
60
40
20
0
Cdc45 WT
Cdc45 2A

Figure S4

A. ChIP of Pol1 at 322 origins sorted by tREP   α factor → HU 60mins

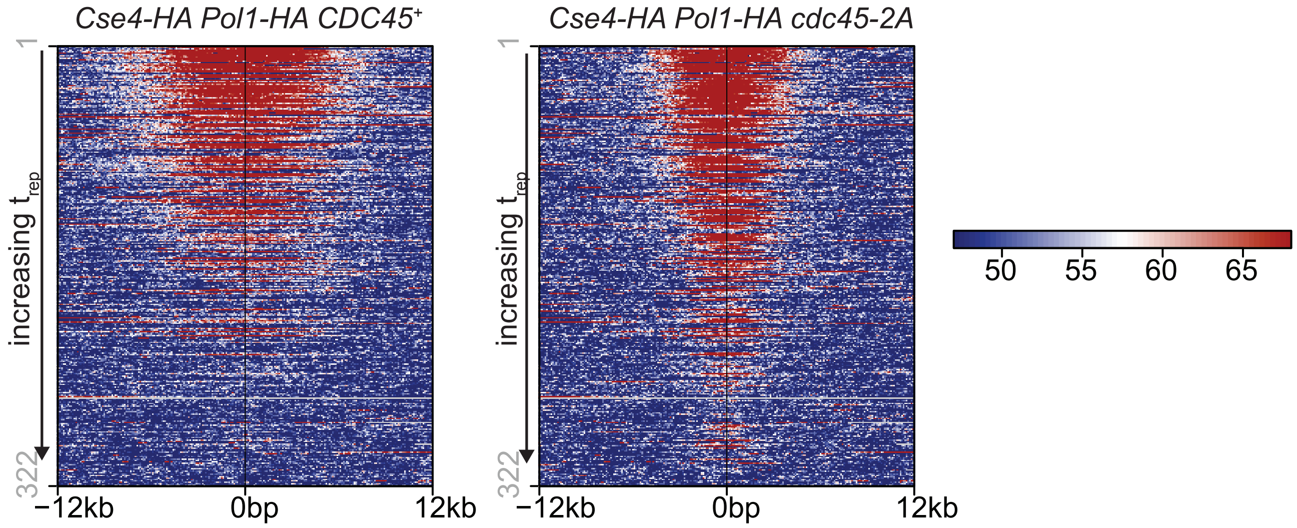

B. Copy number analysis at 322 origins sorted by tREP   α factor → HU 60mins

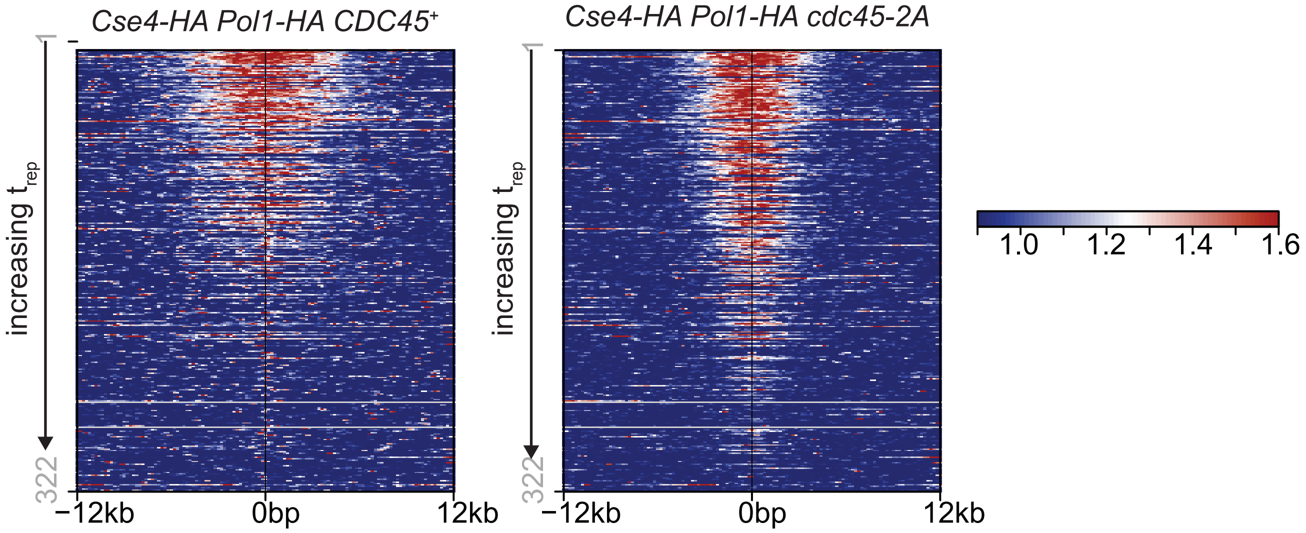

C. Copy number analysis in sld3-A dbf4-A strains   α factor → HU 60mins

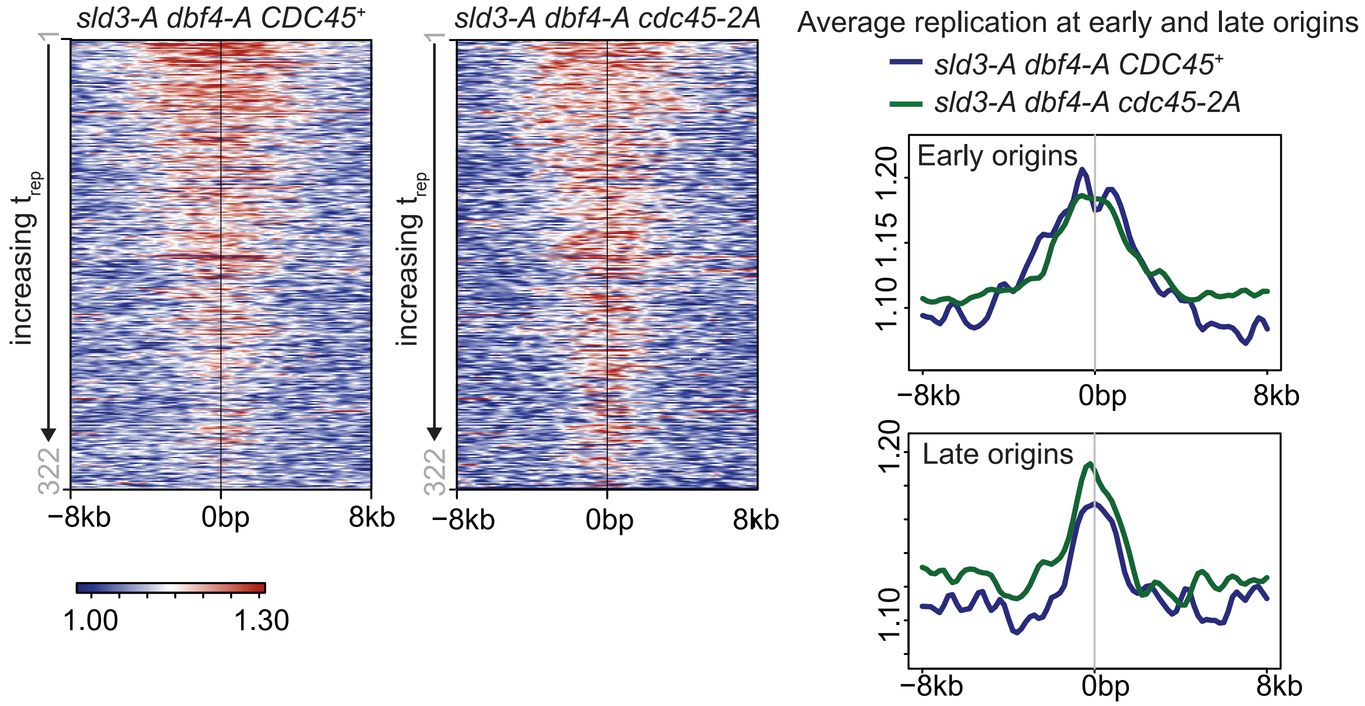

Figure S5

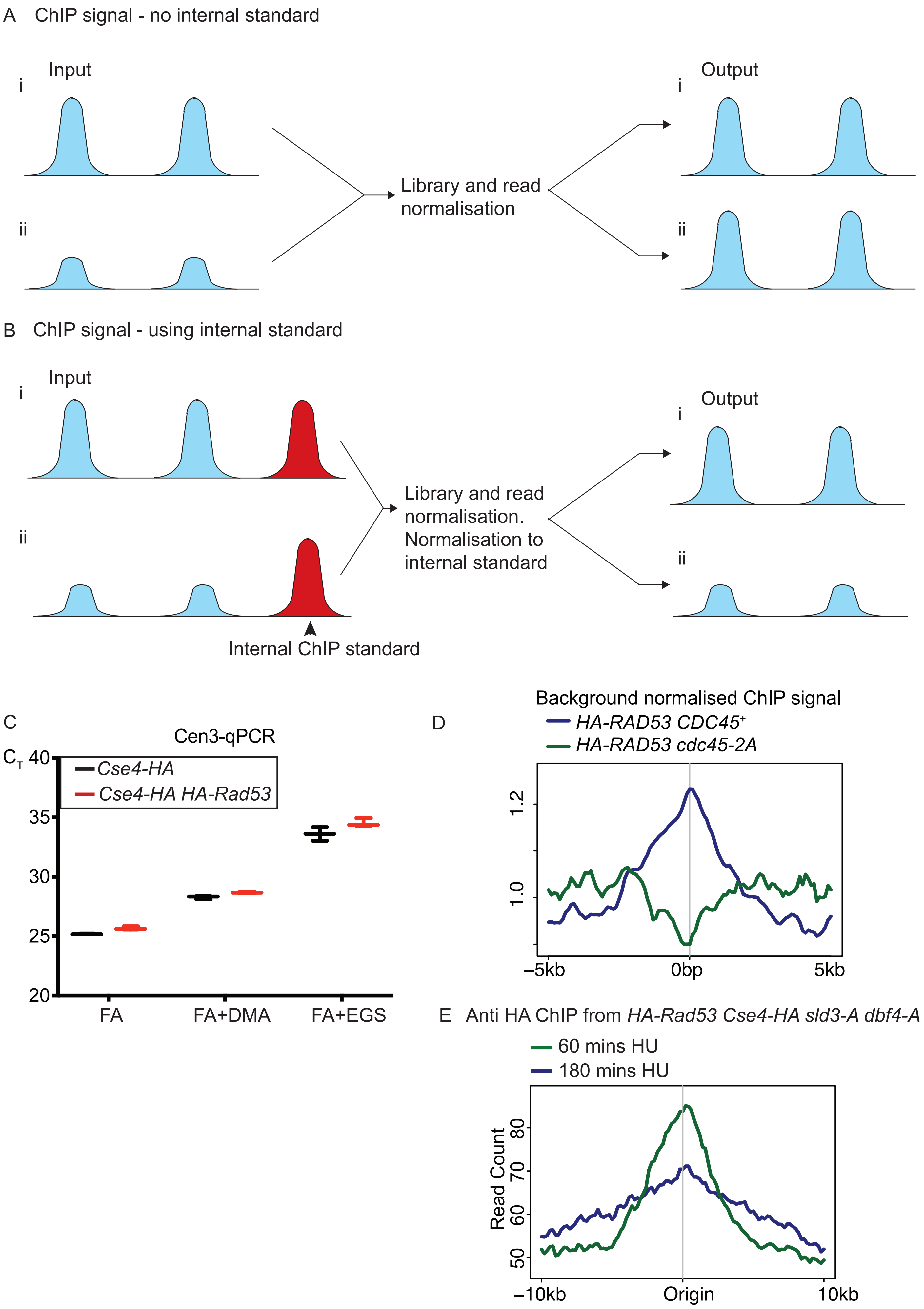
A ChIP signal - no internal standard
Input
i
ii
Library and read normalisation
Output
i
ii
B ChIP signal - using internal standard
Input
i
ii
Internal ChIP standard
Library and read normalisation. Normalisation to internal standard
Output
i
ii
C
Cen3-qPCR
C_T
Cse4-HA
Cse4-HA HA-Rad53
40
35
30
25
20
FA
FA+DMA
FA+EGS
D
Background normalised ChIP signal
HA-RAD53 CDC45+
HA-RAD53 cdc45-2A
1.2
1.0
–5kb
0bp
5kb
E Anti HA ChIP from HA-Rad53 Cse4-HA sld3-A dbf4-A
60 mins HU
180 mins HU
Read Count
80
70
60
50
–10kb
Origin
10kb

Figure S6

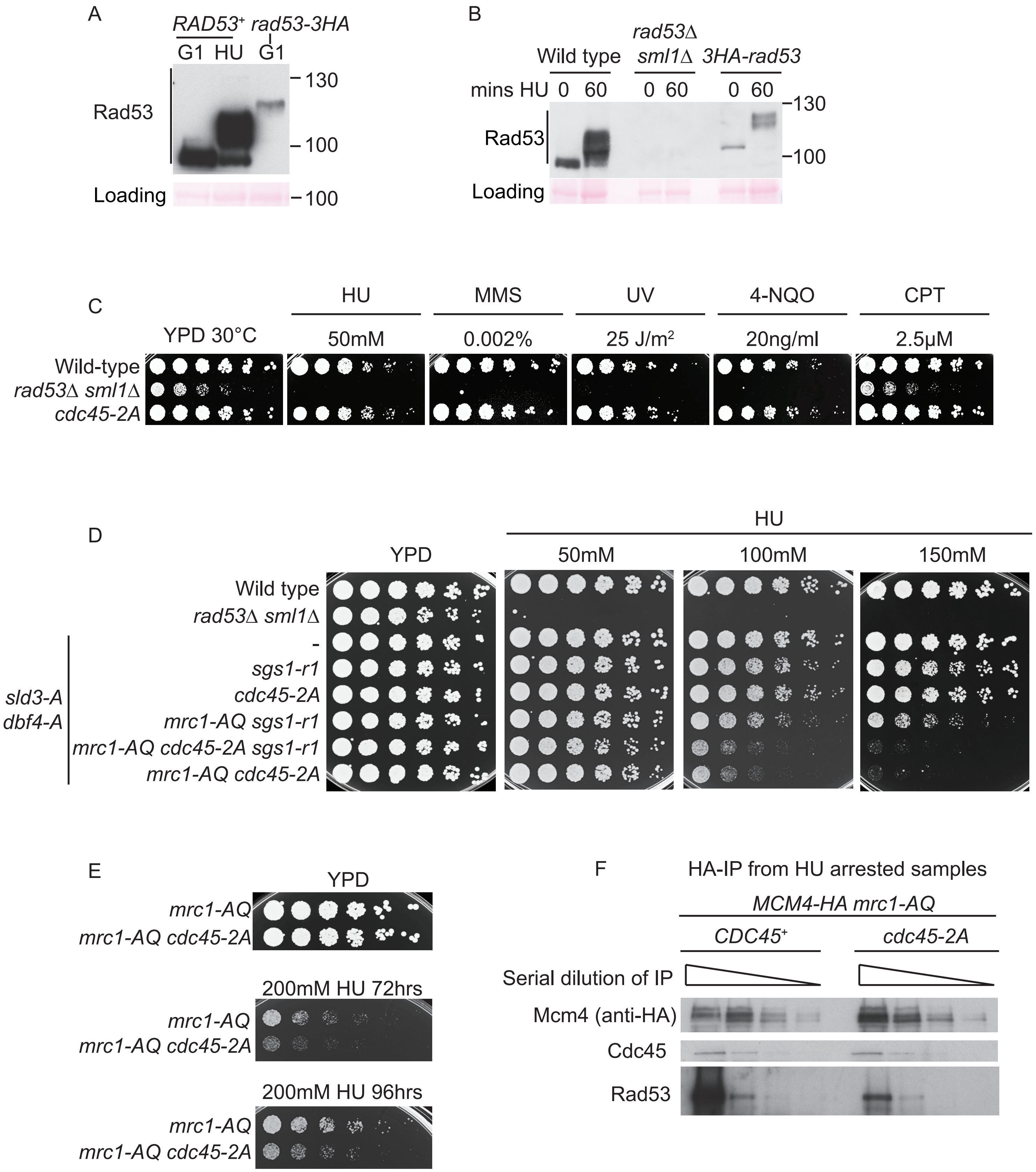
A
RAD53+ rad53-3HA
G1 HU G1
Rad53
130
100
Loading
100
B
Wild type
rad53Δ sml1Δ
3HA-rad53
mins HU 0 60 0 60 0 60
Rad53
130
100
Loading
C
HU MMS UV 4-NQO CPT
YPD 30°C
50mM 0.002% 25 J/m2 20ng/ml 2.5μM
Wild-type
rad53Δ sml1Δ
cdc45-2A
D
HU
YPD 50mM 100mM 150mM
Wild type
rad53Δ sml1Δ
-
sgs1-r1
cdc45-2A
mrc1-AQ sgs1-r1
mrc1-AQ cdc45-2A sgs1-r1
mrc1-AQ cdc45-2A
sld3-A
dbf4-A
E
YPD
mrc1-AQ
mrc1-AQ cdc45-2A
200mM HU 72hrs
mrc1-AQ
mrc1-AQ cdc45-2A
200mM HU 96hrs
mrc1-AQ
mrc1-AQ cdc45-2A
F
HA-IP from HU arrested samples
MCM4-HA mrc1-AQ
CDC45+ cdc45-2A
Serial dilution of IP
Mcm4 (anti-HA)
Cdc45
Rad53

Figure S7

A

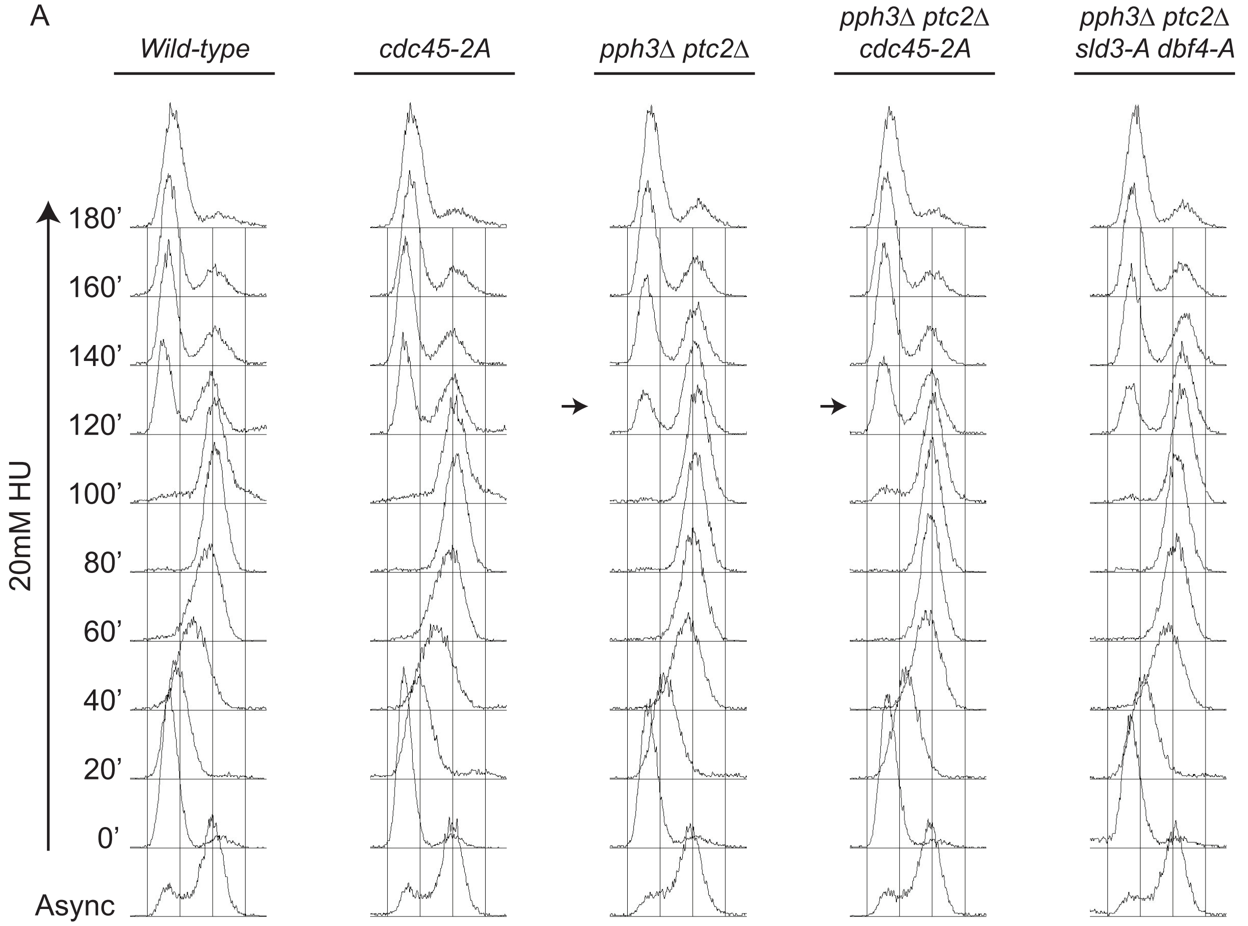
Wild-type
cdc45-2A
pph3Δ ptc2Δ
pph3Δ ptc2Δ cdc45-2A
pph3Δ ptc2Δ sld3-A dbf4-A
20mM HU
180’
160’
140’
120’
100’
80’
60’
40’
20’
0’
Async

B

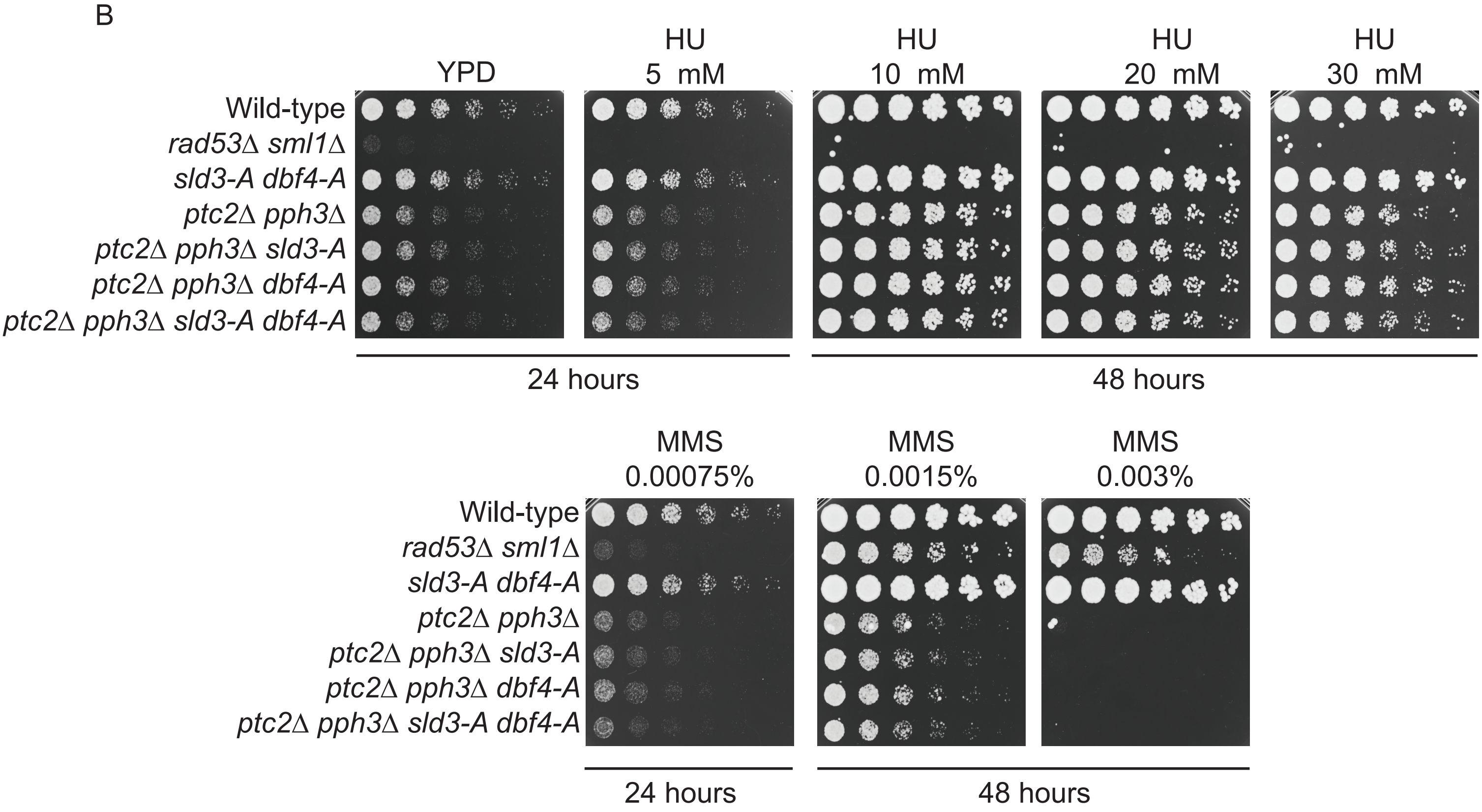
YPD
HU 5 mM
HU 10 mM
HU 20 mM
HU 30 mM
Wild-type
rad53Δ sml1Δ
sld3-A dbf4-A
ptc2Δ pph3Δ
ptc2Δ pph3Δ sld3-A
ptc2Δ pph3Δ dbf4-A
ptc2Δ pph3Δ sld3-A dbf4-A
24 hours
48 hours
MMS 0.00075%
MMS 0.0015%
MMS 0.003%
Wild-type
rad53Δ sml1Δ
sld3-A dbf4-A
ptc2Δ pph3Δ
ptc2Δ pph3Δ sld3-A
ptc2Δ pph3Δ dbf4-A
ptc2Δ pph3Δ sld3-A dbf4-A
24 hours
48 hours

Supplementary figure legends

Figure S1. Phenotypes of Cdc45 mutants, Related to Figure 1 and 2.

A) Flow cytometry (left) and budding index (right) of the indicated Cdc45 mutants released from alpha factor into S-phase at 25°C. The red line indicates the position of the 2N DNA content.

B) Autoradiogram of a Southern blot of an alkaline gel of replication intermediates from strains released from alpha factor into 200mM HU. Southern blot was probed for ARS305.

C) Table of the expected and actual frequency of gentoypes from the indicated genetic cross.

D) as C) (left) and image of tetrads (right) indicating the growth of *rad53* null colonies with and without *cdc45-2A*. Dead spores are *rad53* null and *SML1*$^+$.

E) Western blots of the indicated yeast strains, released from alpha factor (0) into 200mM HU. Loading control is a non-specific band from the HA western blot.

Figure S2. Effect of Cdc45 mutants on the interaction with Sld3 or Rad53, Related to Figure 1 and 2.

A) Flow cytometry (left) and Western blots (right) of the indicated strains arrested at 25°C in nocodazole, then shifted to 37°C before addition of 10μg/ml 4-NQO.

B) Anti-Cdc45 Western blot of the indicated yeast strains, released from alpha factor (α) into 200mM HU for 60 minutes.

C) Yeast-two hybrid growth assay (left) between full length Cdc45 and Sld3 on non-selective (+His) and selective medium (-His). 3-AT is 3-aminotriazole. Right, Western blot of yeast-two hybrid fusion proteins as indicated.

D) Alignment of unstructured acidic loop region of Cdc45 from Saccharomycotina yeast species. Potential FHA interaction TxxD motifs are underlined.

E) Yeast-two hybrid growth assay between full length Cdc45 and Rad53 FHA1 (1-165) on non-selective (-L-T) and selective medium (-L-T-H). WT corresponds to wild type, 1A is T189A, 2A is T189A/T195A. Bottom, Western blot of yeast-two hybrid fusion proteins as indicated.

F) As Figure 1C.

Figure S3. Rad53 is not activated by Cdc45 non-specifically and phosphorylates Cdc45 at T189/T195 *in vitro*. Related to Figure 4.

A) Kinase assay (left) with Rad53, Sld3 C-terminus (530-668) and either BSA or Cdc45. Sld3, Cdc45 and BSA were in 25 fold excess over Rad53. Top; Coomassie stained gel, Middle; autoradiogram. (right) Quantitation of the kinase assay. Error bars are standard deviation, n=3.

B) As A), demonstrating the dependence of Sld3 and Cdc45 phosphorylation on Rad53.

C) Quantitation of the Rad53 phosphorylation of Cdc45 wild type (WT) and Cdc45 T189A/T195A (2A). Cdc45 WT phosphorylation was set to 100%. Error bars are standard deviation, n=4.

Figure S4. Cdc45-2A affects replisome position. Related to Figure 5.

A) Anti-HA (Pol1) ChIP of the indicated strains released from alpha factor (G1) into 200mM HU for 60 mins. Data is presented as a heatmap of sequencing reads normalised to the reads from 500bp either side of all yeast centromeres. The map is centred on 322 of the yeast origins, arranged by increasing t_{rep}.

B) DNA sequencing reads for the input samples before HA-IP from A) were quantified to derive DNA copy number genome-wide. Data was normalised to a G1 sample and is represented as a ratio of S/G1.

C) Left heatmaps as B). Right. Data is represented as a graph of the average signal from 322 origins, centred on the origin. The graphs, separated into early and late firing origins (161 each), demonstrates that average replication amount/position is very similar between strains containing *sld3-A dbf4-A*.

Figure S5. Normalisation of ChIP-seq signal using Cse4-HA. Related to Figure 5.

A) Read/library normalisation from ChIP-seq data can result in distortion of peak heights between samples.

B) Use of internal standard (red) for normalisation can allow direct comparison of peak heights between samples.

C). qPCR of centromeric locus Cen3 from anti-HA ChIP from the indicated strains. ChIP was performed with 3 different crosslinking procedures; formaldehyde alone (FA), FA + dimethyl adipimidate (DMA) or FA + ethylene glycol disuccinate (EGS). Error bars are standard deviation, n=3.

D) Data from Figure 5A, normalised to background reads at an unreplicated genomic locus instead of the Cse4-HA signal around centromeres.

E) Graph representing average signal at 322 origins from anti-HA ChIP reads from strain *HA-Rad53 Cse4-HA sld3-A dbf4-A*. Data was normalised to the Cse4-HA signal, using 500bp either side of all yeast centromeres.

Figure S6. Growth defect of *cdc45-2A* is not exacerbated by the *sgs1-r1* mutant. Related to Figure 6.

A) Western blot of C-terminally tagged Rad53.

B) Western blot of N-terminally tagged Rad53.

C) Growth assay of the indicated strains.

D) as C), the bottom 6 strains are all *sld3-A dbf4-A.*

E) As C).

F) Anti Mcm4-HA IP as in Figure 5D. The IPs were serially diluted 2-fold to give an indication of the relative difference in Rad53-replisome interaction between Cdc45 wild type and *cdc45-2A* strains.

Figure S7. Cdc45-2A suppresses the cell cycle delay of *pph3*Δ *ptc2*Δ strains. Related to Figure 6.

A) Flow cytometry of the indicated strains released from alpha factor (0) into 20mM HU. Arrows highlight the faster progression through the cell cycle of the *pph3*Δ *ptc2*Δ *cdc45-2A* strain.

B) Growth assay of the indicated strains.
